# Temporal Heterogeneity of Factors Associated with Intra-ICU Mortality: A Formal Interaction Analysis of the Multi-Centre Silesian ICU Registry (2010–2020)

**DOI:** 10.3390/jcm15187013

**Published:** 2026-09-10

**Authors:** Piotr S. Liberski, Krzysztof Żerdziński, Michał Gałuszewski, Michał Skrzypek, Łukasz J. Krzych

**Affiliations:** 1 Department of Anesthesiology, Intensive Therapy and Periopertive Medicine, Department of Acute Medicine, Faculty of Medical Sciences in Zabrze, Medical University of Silesia in Katowice, 41-800 Zabrze, Poland; pliberski@sum.edu.pl (P.S.L.); lkrzych@sum.edu.pl (Ł.J.K.); 2 Department of Anesthesiology and Intensive Care, Upper-Silesian Medical Center, 40-635 Katowice, Poland; 3 Students Department “#Intensywna_Po_Godzinach”, Department of Acute Medicine, Faculty of Medical Sciences in Zabrze, Medical University of Silesia in Katowice, 41-800 Zabrze, Poland; s86715@365.sum.edu.pl; 4 Department of Biostatistics, Faculty of Public Health in Bytom, Medical University of Silesia in Katowice, 41-902 Bytom, Poland; mskrzypek@sum.edu.pl

**Keywords:** intensive care, mortality, prognosis, temporal trends, effect modification, interaction, ICU registry, odds ratio, false discovery rate, calibration drift

## Abstract

**Background:** Severity-of-illness scores and registry-derived risk models assume that the association between a baseline characteristic and death is stable over time. **Methods:** A retrospective analysis of 25,344 admissions (2010–2020) to 34 intensive care units in the Silesian Registry of Anaesthesiology and Intensive Care, with unchanged variable definitions and intra-ICU death as the outcome, was conducted. For each of 48 characteristics, a characteristic-by-time interaction was tested by likelihood ratio under four implementations of time-chronological quintiles, fixed calendar periods, linear and spline continuous time, each independently Benjamini–Hochberg corrected. **Results:** Twenty-two of 48 characteristics showed significant heterogeneity in the primary quintile analysis (*q* < 0.05), with 11 under all four. Adjustment for centre left 12 significant, 6 of which also survived adjustment for case mix. An adjusted model retained discrimination out of period but over-predicted risk in the final quintile. **Conclusions:** For nearly half of the characteristics, the crude association with intra-ICU death is not temporally stable, although most of the movement tracks a changing population. Out of period, a fixed model loses the level of predicted risk, not the weighting of its predictors-level rather than structural drift. Multi-year registry analyses should test temporal homogeneity rather than assume it.

## 1. Introduction

By virtue of the severity of their critical illness, patients admitted to the intensive care unit (ICU) carry a substantial risk of death, and the objective stratification of that risk is a long-standing task of critical-care medicine. Severity-of-illness scoring was introduced precisely to meet this need. By translating routine physiological and clinical data into a quantified probability of death, such scores allow clinicians to stratify acutely ill patients prognostically, compare critical-care resource use and the efficacy of intensive care across institutions and over time, and support patient stratification in clinical research [1]. The most widely adopted instruments—the Acute Physiology and Chronic Health Evaluation (APACHE), the Simplified Acute Physiology Score (SAPS) and the Sequential Organ Failure Assessment (SOFA)—have become embedded in ICU practice and research worldwide [1,2,3].

Although these scores perform acceptably at the population level and remain a fixture of daily practice, their long-term application has exposed important limitations [4]. Because every such model is derived from a historical development cohort, its predictions can diverge from observed outcomes as the case mix and treatment of critically ill patients change—a phenomenon termed calibration drift, which has been documented repeatedly for clinical prediction models and tends to worsen as the interval from model derivation lengthens [5,6]. Divergence also arises across settings. Predicted and observed mortality frequently differ when a score developed elsewhere is applied to a local population, which is why external and local validation are regarded as indispensable [7]. In the Silesian ICU population specifically, observed mortality has been shown to be substantially lower than that predicted by the APACHE II system [8], and the wider question of how much clinical value these scores retain in contemporary intensive care remains actively debated [4].

A tacit assumption underlying most registry-based analyses is that the association between a given baseline characteristic and death is itself stable over time. This premise is questionable, because the critically ill population evolves continuously. Advances in oncology and haematology have improved the survival of critically ill cancer patients [9]. Outcomes in conditions such as severe sepsis have improved markedly across successive years [10], and shifting admission practices have altered the very spectrum of diagnoses reaching the ICU [11]. Long-running registry data make the same point directly. Across seventeen years of Finnish intensive care, patients treated for traumatic brain injury became older and more comorbid while their mortality fell [12]. Where clinical prediction models have been examined over time, both mechanisms of change have been observed. In some settings, calibration drift has been attributed chiefly to a changing case mix [6], whereas in others, the weight of individual predictors themselves has shifted, with some factor–outcome associations strengthening and others weakening [13]. Despite this, most epidemiological evaluations continue to treat multi-year cohorts as temporally homogeneous—an approach that risks conflating these mechanisms and masking clinically meaningful, era-dependent change. How the strength of individual factor–outcome associations evolves over extended periods, therefore, remains insufficiently characterised and is only rarely subjected to a formal statistical test.

The Silesian Registry of Anaesthesiology and Intensive Care provides a rare opportunity to examine this question directly. Established as a long-term, multi-centre database governed by standardised, unchanging variable definitions and in continuous operation across the study period, it has recorded the same clinical descriptors for critically ill patients throughout the region [14], allowing for the association between each baseline factor and death to be compared on a like-for-like basis between distinct chronological periods.

The primary aim of this study was to test formally—rather than to describe—whether the association between individual baseline characteristics and intra-ICU death changed over the study period. For each candidate characteristic, we tested a characteristic-by-time interaction, repeated the test under four implementations of time—chronological quintiles, fixed calendar periods, linear continuous time, and spline continuous time—so that no single choice of temporal stratification could drive the conclusion, and corrected for multiplicity within each family of tests. Because every primary estimate is marginal—a crude odds ratio from a single-predictor model—a secondary aim was to quantify how much of any temporal movement is attributable to the two obvious alternatives to a genuine change in prognostic meaning: an evolving case mix and an evolving set of contributing centres. A third aim was to determine whether a model fitted on these characteristics loses accuracy when applied to a period on which it was not fitted and, if so, whether what it loses is the overall level of predicted risk or the relative weighting of its predictors.

## 2. Materials and Methods

### 2.1. Study Design and Setting

This was a retrospective, observational, multi-centre study based on prospectively collected data from the Silesian Registry of Anaesthesiology and Intensive Care, a regional database covering 34 multi-profile ICUs, approximately one-third of those operating in the Silesian region of Southern Poland. Three of the 34 units lie just outside the Silesian voivodeship and contributed 361 admissions in total, all in the first quintile. Throughout the study period, data were recorded according to standardised protocols, with uniform variable definitions applied across all participating centres. Reporting follows the STROBE recommendations for observational studies [15].

### 2.2. Data Source

The Silesian ICU Registry is a long-term, continuously maintained, multi-centre database whose design and operating principles have been described previously [8,14]. It records patients’ pre-admission health burden, their clinical status on admission, the primary and direct reasons for ICU admission, and the course and outcome of ICU treatment. Data are entered by the attending physician upon completion of each ICU stay (discharge or death) using a predefined data dictionary, in which the majority of fields are captured as standardised categorical entries. The database contains no data enabling patient identification. Entries are checked for internal consistency but are not routinely audited against the primary medical records at the registry level.

### 2.3. Study Period, Participant Selection and Analytical Cohort

The raw registry export made available for this analysis contained 25,465 admission records. Two pre-specified cleaning rules were then applied. First, records with internally inconsistent admission and discharge dates were excluded: negative length of stay (*n* = 8) and length of stay exceeding 365 days (*n* = 11). In every case, the pattern was consistent, with a one-year data-entry error in the admission or discharge date, verified against the timestamp of the database entry. Second, because the registry became operational on 3 September 2010, the 102 records with an earlier admission date were excluded (84 admitted in 2007–2009 and 18 between 19 July and 2 September 2010). The 2007–2009 records originated from a single centre and were entered into the database in 2017, and their discharge dates also fell in 2007–2009, identifying them as a retrospective historical import rather than data-entry errors. Restricting the cohort to admissions from the date the registry became operational resolves the discrepancy between the raw export and the published description of the registry [14]. Whether including this historical import instead changes the primary result is tested directly as a sensitivity analysis (Section 2.7.7).

**Figure 1:**
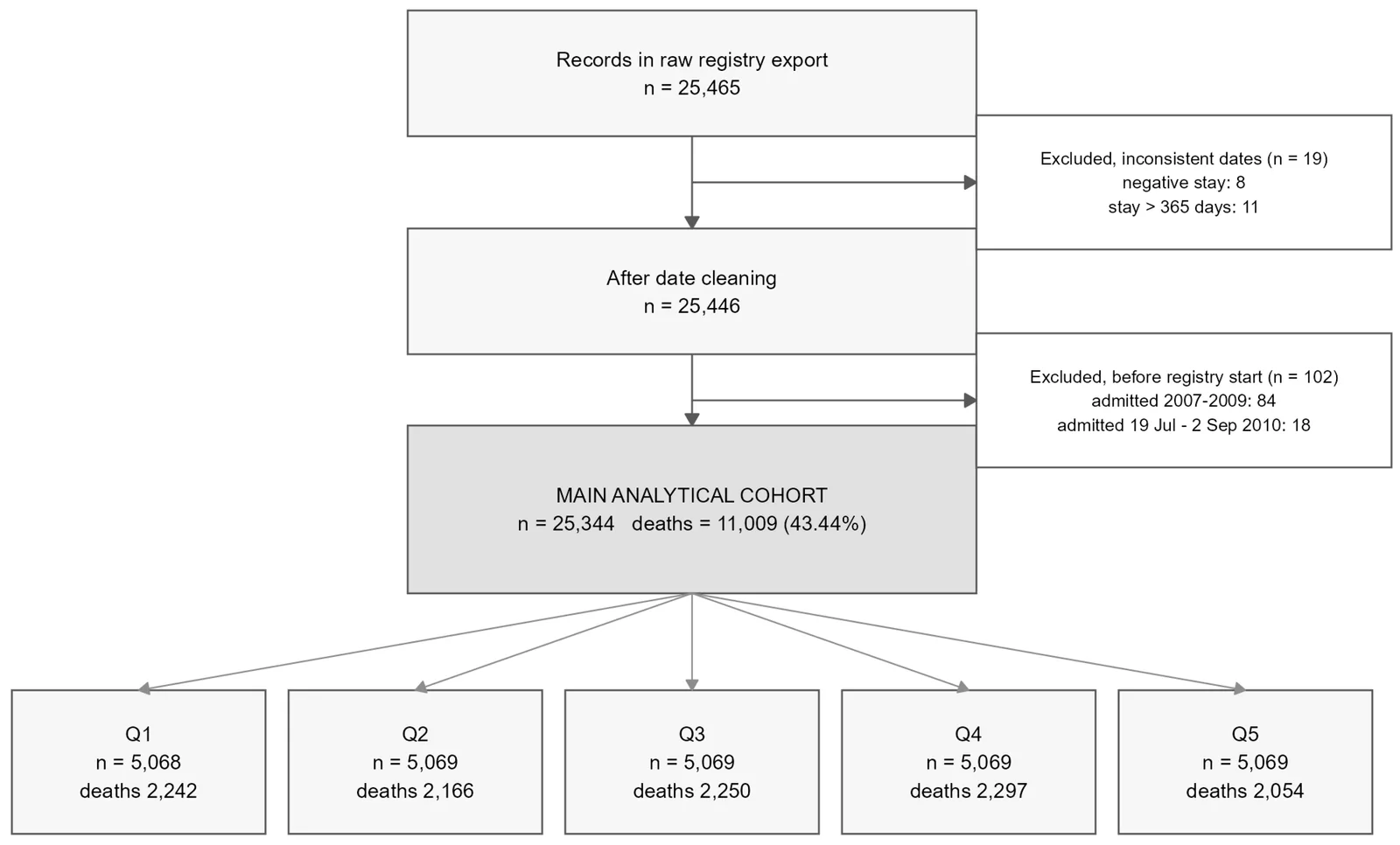
Selection of the analytical cohort. Records with internally inconsistent admission and discharge dates were excluded (negative length of stay, *n* = 8; length of stay exceeding 365 days, *n* = 11, each consistent with a one-year data-entry error verified against the timestamp of the database entry). Admissions preceding the operational start of the registry on 3 September 2010 were also excluded (*n* = 102). The resulting cohort was divided into five equally sized chronological quintiles by admission order.

The resulting analytical cohort comprised 25,344 admissions between 3 September 2010 and 25 January 2020 (Figure 1). No further exclusion criteria were applied. A complete listing of the 121 excluded records is available to the reviewers and to the registry custodian but is not published, because the combination of centre and exact admission date is potentially identifying. The export supplied to the investigators had been restricted to admissions with a complete clinical record. Whether the registry additionally holds incomplete records, and how many, could not be established from the export, and this is stated as a limitation (Section 4.5). The unit of analysis is the ICU admission, not the patient. The database does not carry a reliable patient identifier across hospitalisations, so admissions could not be clustered by patient (Section 4.5).

### 2.4. Outcome Definition

The primary outcome was all-cause intra-ICU death, defined as death occurring during the index ICU admission. Patients who survived to ICU discharge were classified as survivors, regardless of their subsequent clinical course or discharge destination. This binary endpoint served as the dependent variable in all models.

Two outcome-coding issues are declared explicitly. First, 921 records (3.6% of the cohort) carry a general outcome code whose exact meaning could not be confirmed with the registry custodian. All 921 carry the detailed outcome code for “discharged with improvement”, a general condition recorded as “good”, and a transfer destination other than death, and they were, therefore, classified as survivors. They occur only in the fourth (*n* = 165) and fifth (*n* = 756) quintiles and originate from two centres between 2016 and 2019. Their influence on the results is quantified in a dedicated sensitivity analysis (Section 2.7.7). Second, one record contained a contradictory outcome. Four of the five outcome-related fields (general outcome, general condition, neurological status and transfer destination) indicated death, while the detailed outcome field indicated discharge with improvement. This record was counted as a death. The correction was implemented in a derived variable. The source file was not modified, and the affected record is flagged in the analysis code (Section 2.7.8).

### 2.5. Candidate Characteristics

Forty-eight characteristics recorded in the registry’s standardised fields were analysed. For clarity of presentation only, they are grouped into four domains defined by their temporal relationship to admission. The demographic and administrative variables were age, sex, type of admission (medical versus surgical) and episode type (first ICU admission versus re-admission). Pre-admission comorbidities were coronary artery disease, chronic heart failure, arterial hypertension, disseminated atherosclerosis, chronic respiratory failure, extreme obesity, cachexia, alcoholism, diabetes mellitus, chronic renal failure, previous CNS stroke, chronic neurological disease, autoimmune or systemic disease, malignancy, and the residual “other” and “none” categories. The primary reasons for ICU admission were acute respiratory failure, exacerbation of chronic respiratory failure, the composite of the two, primary circulatory failure, multi-organ failure, shock, cardiac arrest, primary disturbance of consciousness, post-surgical state, traumatic multi-organ injury, head injury, acute pancreatitis, acute neurological condition, poisoning, severe metabolic disturbance, infection, severe sepsis, the composite of infection or severe sepsis, and a residual “other” category. Clinical status at the moment of admission comprised respiratory failure, circulatory failure, renal failure, disturbance of consciousness, metabolic disturbances, multi-site traumatic injury, requirement for enhanced monitoring, non-invasive ventilation and a residual “other” category. ICU length of stay was not treated as a candidate characteristic because it is determined after admission and reflects the course of treatment rather than the baseline profile. This grouping organises the presentation only. It does not constitute an analytical stratification.

Forty-seven of the 48 characteristics were complete. Type of admission was missing for 1319 records (5.2%), and completeness improved over the study period (8.6% missing in the first quintile against 3.6% in the last). The primary analysis of this variable is complete-case. A pre-specified re-test treating “not recorded” as an explicit third category, so that no record is dropped, is reported alongside it (Section 3.8).

### 2.6. Parametrisations of Time

Calendar time was represented in four ways, so that the conclusion would not depend on a single arbitrary stratification.

First, as the primary analysis, the cohort was divided into five consecutive equally sized chronological quintiles by admission date: Q1 (3 September 2010–13 January 2012, *n* = 5068), Q2 (13 January 2012–8 May 2013, *n* = 5069), Q3 (8 May 2013–1 December 2014, *n* = 5069), Q4 (1 December 2014–16 November 2016, *n* = 5069) and Q5 (16 November 2016–25 January 2020, *n* = 5069). Because the quintiles are equal in size rather than in duration, the calendar span of each differs, and the number of deaths per stratum ranges from 2054 to 2297. Statistical power is, therefore, similar but not identical across strata. Equal size was chosen so that no stratum would be dominated by the sparse early years of the registry.

Second, the cohort was divided into fixed calendar periods of approximately two years (2010–2011, 2012–2013, 2014–2015, 2016–2017 and 2018–2020), which addresses the concern that equally sized strata partly confound calendar time with the rate of registry accrual. Third, calendar time was modelled as a continuous linear variable in years since 1 January 2010. Fourth, the same continuous time variable was modelled as a natural cubic spline with three degrees of freedom (interior knots at 4 December 2012 and 8 July 2015), to allow a non-linear trajectory. The anchor date was fixed a priori and identically in every cohort variant so that the coefficients remain comparable.

### 2.7. Statistical Analysis

#### 2.7.1. Descriptive Statistics

Categorical variables are summarised as counts and percentages and continuous variables as medians with the first and third quartiles. Comparisons between survivors and non-survivors used Pearson’s chi-squared test for categorical variables and the Wilcoxon rank-sum test for continuous variables. Cohort composition is reported for the whole cohort and separately within each quintile and each fixed calendar period.

#### 2.7.2. Crude Associations

For each candidate characteristic, a separate univariable binary logistic regression model was fitted with intra-ICU death as the dependent variable, and the association expressed as a crude odds ratio with a 95% confidence interval. The estimand throughout the primary analysis is, therefore, the marginal (unadjusted) association between a characteristic and death within the registry population of that period. Crude odds ratios were computed for the whole cohort and separately within each temporal stratum. Because the characteristics are measured on different scales and with different reference categories, age is expressed per year. All other characteristics are binary. The numerical magnitude of a crude odds ratio is not a measure of clinical importance or of predictive contribution, and no ranking of characteristics by odds ratio is used as evidence in this analysis.

#### 2.7.3. Formal Test of Temporal Change (Primary Analysis)

For each characteristic, temporal change was tested formally by comparing two nested logistic models: a main-effects model containing the characteristic and the temporal stratum, and a model additionally containing their interaction. The two were compared by a likelihood-ratio test, which carries four degrees of freedom for the five-level quintile and fixed-period stratifications, one degree of freedom for linear continuous time and three for the spline. A significant test indicates that the association between the characteristic and death differs between periods. It makes no assumption of monotonicity, which matters because most observed trajectories are not monotonic. A test for trend was, therefore, not used as the primary test. The same procedure was repeated in each of the four parametrisations of time, giving four independent families of 48 tests each.

#### 2.7.4. Multiple Comparisons

Multiplicity was handled by controlling the false discovery rate with the Benjamini–Hochberg procedure [16] applied separately within each of the four families of 48 tests. Families were never pooled into a single correction. False-discovery-rate control rather than family-wise control was chosen because the analysis is a screen across all recorded characteristics rather than a confirmatory test of one pre-specified hypothesis, and because a Bonferroni-type correction across 192 tests would have been strongly conservative in the presence of correlated characteristics. Corrected values are reported as *q*. Findings are graded explicitly by the number of parametrisations under which they reach *q* < 0.05, and results significant in one parametrisation only are treated as exploratory.

#### 2.7.5. Case-Mix, Centre Composition and Adjusted Analyses

Because a crude odds ratio depends jointly on the characteristic and on the composition of the population against which it is contrasted, two alternative explanations for any temporal movement were tested rather than merely acknowledged.

Collinearity among candidate characteristics was quantified before any adjusted model was considered by the matrix of pairwise correlations (Pearson, phi or point biserial as appropriate to the variable types) and by variance inflation factors from an auxiliary model containing all non-composite characteristics. A decision rule was fixed in advance. An adjusted model would be fitted only if the maximum variance inflation factor was below 5 and fewer than five characteristics exceeded 2.5. Where this rule was met, an adjusted model was fitted after removing composite and residual variables and the less informative member of each pair correlated above 0.5. Each characteristic-by-quintile interaction was then re-tested in the adjusted model with the same Benjamini–Hochberg correction. This analysis is supplementary; it asks a different question from the primary analysis and does not replace it.

Centre composition was addressed in two ways that fail in opposite directions, so that their agreement is informative. Adding centre as a fixed effect over-adjusts because centres leave the registry over time and the fixed effect, therefore, absorbs part of the genuine temporal signal. Restricting the analysis to the ten centres contributing to all five quintiles (a balanced panel, *n* = 15,722) loses power because it discards 38% of the cohort. Both were run and reported. Standard errors were not clustered because the database carries no reliable patient identifier across hospitalisations, and a likelihood-ratio test does not accommodate a robust variance matrix without being replaced by a different test.

Characteristics whose temporal heterogeneity is significant in the crude analysis and remain significant after adjustment both for case mix and for centre are reported as findings robust to the measured adjustments: heterogeneity not explained by the recorded case mix or by centre composition. Because adjustment can only use recorded characteristics, this is not evidence that the underlying prognostic meaning has changed. Residual confounding by unrecorded severity of illness cannot be excluded (Section 4.5).

#### 2.7.6. Temporal Validation of the Adjusted Model

To separate a change in the overall level of risk from a change in the relative weighting of the characteristics, the adjusted model was subjected to rolling-origin temporal validation in the manner recommended for prediction models [17]. The model was refitted on quintiles Q1 to Qk and evaluated on the next unseen quintile for k = 1 to 4. Discrimination was measured by the area under the receiver-operating characteristic curve, and calibration by the calibration intercept and slope obtained by regressing the observed outcome on the linear predictor in the unseen period. A calibration slope near unity with a non-zero intercept indicates that the level of predicted risk has drifted while the relative weighting of the characteristics has been preserved. A slope departing from unity indicates that the weights themselves no longer apply.

#### 2.7.7. Sensitivity Analyses

Three sensitivity analyses, each repeating the full primary analysis, were pre-specified. The first excluded the 921 records with an unconfirmed outcome code (*n* = 24,423), keeping the quintile boundaries of the main cohort so that only the effect of removal is tested. The second applied the original 180-day length-of-stay rule instead of the 365-day rule (*n* = 25,338), isolating the effect of the date-cleaning rule alone. The third included the pre-2010 historical import excluded from the main cohort (Section 2.3), recomputing quintiles on the full 2007–2020 export under the 180-day rule (*n* = 25,440) to test whether excluding that import drives the primary result. This analysis does not redefine the analytical cohort used elsewhere in this manuscript. For each, the number of characteristics reaching *q* < 0.05 and the number of conclusions changed relative to the primary analysis are reported.

#### 2.7.8. Software and Reproducibility

All analyses were performed in R version 4.6.1 (R Foundation for Statistical Computing, Vienna, Austria), with descriptive tables produced using the gtsummary package [18]. The analysis was implemented as sixteen scripts run in a fixed order under a locked package environment. Each script terminated with assertions of its own control numbers, causing the pipeline to halt rather than proceed silently if any number drifted. A separate audit script independently recomputed 49 invariants from the cohort file. The code and its execution logs are available from the corresponding author upon reasonable request.

### 2.8. Ethical Considerations

Owing to its retrospective and registry-based nature, this study was not considered a medical experiment. Ethical review and approval by the Bioethics Committee of the Medical University of Silesia in Katowice were therefore waived, in accordance with the applicable legal provisions (Act of 5 December 1996, Journal of Laws 2011 No. 277, item 1634, as amended; decision no. KNW/0022/KB/122-2/14, dated 13 May 2014). The study was conducted in accordance with the principles of the Declaration of Helsinki.

### 2.9. Use of Generative Artificial Intelligence

Generative artificial intelligence was used in the preparation of this manuscript and the revised analysis, and its role is declared in full here.

Large language models (Claude, Anthropic: Sonnet 5, Opus 5, Fable 5 and Opus 4.8; ChatGPT, OpenAI: GPT 5.6 Terra and Sol) were used to plan and to write the R code implementing the statistical analysis reported in this revision, and to draft manuscript text, section headings, figure and table captions, and to perform language editing. The analysis plan, the choice of estimand, the pre-specified decision rules and every substantive interpretation were determined by the authors. The models did not select results, did not choose which findings to report, and had no access to the source data other than through the code the authors reviewed and executed. Gemini 3.1 Pro (Google) was used to assist with language editing and to produce the graphical abstract from an author-prepared brief. All numerical values shown in the graphical abstract were taken from the analyses reported in this manuscript and were checked against them by the authors. The model produced no quantitative content of its own.

Because a substantial part of the analysis code was machine-drafted, verification was built into the pipeline rather than performed only at the end. Each script asserts its own control numbers, and an independent audit script recomputes the principal quantities from the cohort file (Section 2.7.8). The code and its execution logs are available from the corresponding author upon reasonable request (Section 2.7.8). All AI-assisted content—text, code and figures—was critically reviewed, edited and validated by the authors, who confirm that no data were fabricated or misrepresented and who take full responsibility for the content of this publication.

## 3. Results

### 3.1. Analytical Cohort and Baseline Characteristics

Of the 25,465 records in the raw registry export, 19 were excluded for internally inconsistent dates and 102 for an admission date preceding the operational start of the registry, leaving an analytical cohort of 25,344 admissions between 3 September 2010 and 25 January 2020 (Figure 1). Intra-ICU mortality was 43.4% (*n* = 11,009), and 14,335 admissions (56.6%) ended in discharge from the ICU.

The median age was 66 years (interquartile range 56–76), and non-survivors were about five years older than survivors (median 69 versus 64 years; *p* < 0.001), while sex did not differ between outcome groups (*p* = 0.76). Medical admissions predominated among non-survivors, whereas surgical admissions predominated among survivors; the acute organ-failure syndromes recorded at admission showed the widest gaps between the outcome groups of any characteristic. The chronic-disease profile was that of a contemporary Polish ICU population, dominated by arterial hypertension, coronary artery disease, chronic heart failure and disseminated atherosclerosis. Two comorbidities ran against the general pattern of higher prevalence among non-survivors; malignancy and chronic neurological disease were both more common among survivors, a reversal discussed in Section 4.2. Full baseline characteristics stratified by outcome are given in Table 1.

### 3.2. Change in Cohort Composition and in Centre Participation over the Study Period

**Figure 2:**
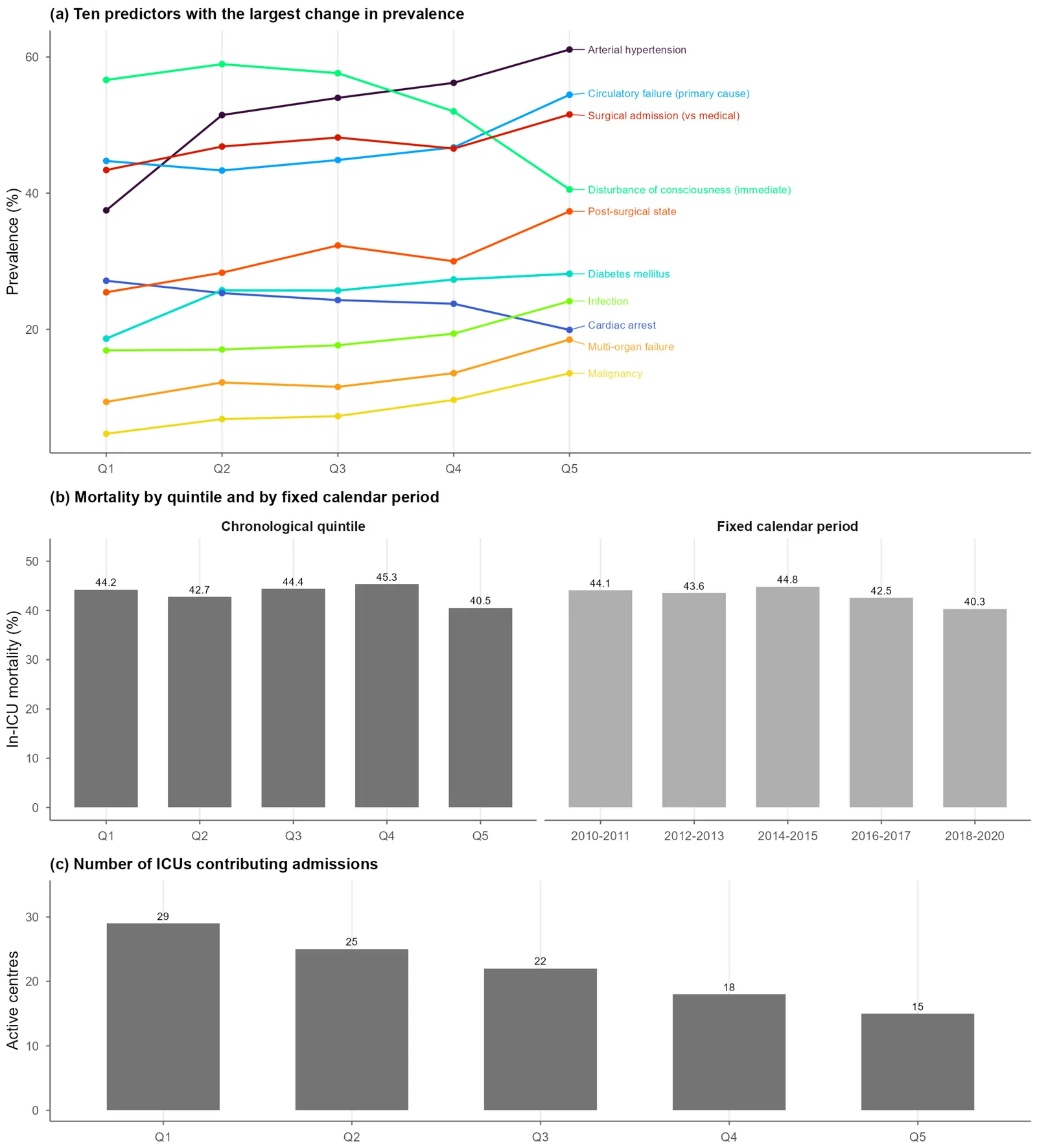
Change in cohort composition over the study period. (**a**) The ten characteristics whose prevalence changed most between the first and the last quintile. (**b**) Intra-ICU mortality by chronological quintile and by fixed calendar period. (**c**) Number of intensive care units contributing at least one admission in each quintile. Together, these panels quantify the compositional change that accompanies, and may partly explain, the drift in crude odds ratios reported in Section 3.4.

Cohort composition changed substantially across the five quintiles (Table 2, Figure 2a). The largest shifts were concentrated in the cardiovascular and acute-organ-failure characteristics discussed in Section 4.2. The recorded prevalence of arterial hypertension rose by about three-fifths in relative terms (37.5% to 61.1%) and malignancy nearly tripled (4.7% to 13.5%, a relative increase of 191%), while cardiac arrest as a reason for admission became less frequent (27.1% to 19.9%), even as its crude odds ratio was higher in Q5 than in Q1 (Section 3.6). The median age rose from 65 to 67 years, and intra-ICU mortality was 44.2% in the first quintile and 40.5% in the last, with on-monotonic intermediate values (42.7%, 44.4%, 45.3%). The same net movement was observed across fixed calendar periods (Figure 2b).

Centre participation was not constant. All 34 centres and their admission counts by quintile are listed in Table 3. The number of ICUs contributing at least one admission fell from 29 in the first quintile to 15 in the last, and recruitment became correspondingly concentrated. The share of admissions contributed by the five largest contributors rose from 35.8% to 58.8% and the Herfindahl–Hirschman index of concentration from 0.049 to 0.097 (Figure 2c). Only ten centres contributed to all five quintiles, together accounting for 15,722 admissions (62% of the cohort), while eleven contributed to one or two quintiles only. This drift in the contributing population is the principal alternative explanation for any apparent temporal change in a crude association, and it is tested directly in Section 3.8.

### 3.3. Crude Associations in the Whole Cohort

In the whole cohort, the highest crude odds ratios for death were observed for variables describing clinical status at the moment of admission and the acute reasons for admission, with several exceeding 2.85. Pre-admission comorbidities were associated with more moderate crude odds, with the strongest below two. The characteristics most strongly associated with lower crude odds of death—non-invasive ventilation, poisoning and traumatic injury on admission—each reflect a less acutely compromised presentation at admission rather than a causal effect that lowers risk. Of the 48 characteristics, 43 reached *p* < 0.05. Female sex, chronic respiratory failure, extreme obesity, autoimmune or systemic disease and the residual “other pre-admission condition” category did not. Crude estimates for all characteristics are given in Table 4. These are marginal associations, not adjusted for one another, and their relative magnitudes should not be read as a hierarchy of clinical importance, because the characteristics are measured on different scales and with different reference categories.

### 3.4. Formal Test of Temporal Heterogeneity (Primary Analysis)

**Figure 3:**
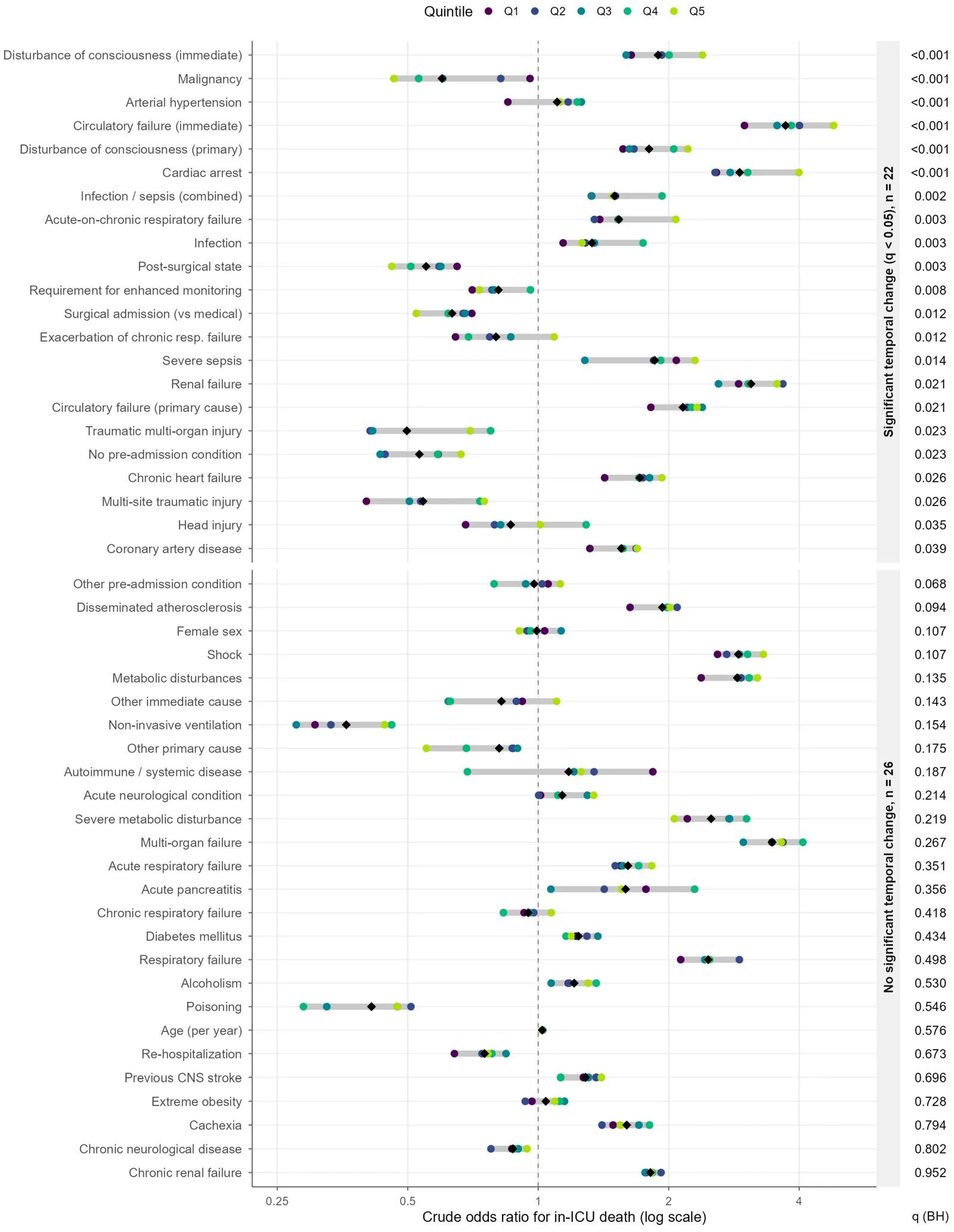
Temporal landscape of crude associations with intra-ICU death for all 48 candidate characteristics. Each row shows one characteristic: the black diamond is the crude odds ratio in the whole cohort, the coloured points are the crude odds ratios within each chronological quintile, and the grey bar spans their range. Characteristics are ordered by the *q* value of the likelihood-ratio test of the characteristic-by-quintile interaction (4 degrees of freedom, Benjamini–Hochberg correction across the 48 tests) and split into those reaching *q* < 0.05 and those not. The dashed line marks an odds ratio of 1. Odds ratios are on different scales across characteristics (age is per year; all other characteristics are binary), so vertical position reflects the evidence for temporal change, not clinical importance.

In the primary analysis, 24 of the 48 characteristics showed heterogeneity in their association with death across the five chronological quintiles at an uncorrected *p* < 0.05, and 22 remained significant after Benjamini–Hochberg correction within this family of 48 tests (*q* < 0.05). The full results, including the crude odds ratio in each quintile, the likelihood-ratio statistic and the corrected *q* value, are given in Table 5 and displayed in Figure 3. No characteristic triggered a sparsity, convergence or separation flag, and every interaction model converged.

Twenty-one of the 22 significant trajectories were non-monotonic. Malignancy was the only one of those 22 whose crude odds ratio moved consistently in one direction across all five quintiles. Monotonic trajectories are not absent elsewhere in the analysis—shock on admission, which is not significant under this stratification, is one—but among the characteristics for which heterogeneity was demonstrated, malignancy is the single case. This is the empirical justification for using an interaction test rather than a test for trend as the primary analysis. A test for trend would have missed most of the heterogeneity actually present, while directional language of the kind a descriptive reading invites would have been unsupportable for the majority of these characteristics.

### 3.5. Robustness of the Temporal Signal Across Parametrisations of Time

The primary result did not depend on the choice of temporal stratification, although its extent did (Table 6). Under fixed two-year calendar periods, 24 of 48 characteristics reached *q* < 0.05, and the two stratifications agreed on 75.0% of characteristics (17 significant under both, 19 under neither, 5 under quintiles only and 7 under fixed periods only). Modelling calendar time as a continuous linear term identified 14 of 48, and as a natural spline with three degrees of freedom, 21 of 48. The discrepancy between the last two is informative. Nine characteristics were significant under the spline but not under the linear term, meaning that their association with death changed in a way that is real but is not a steady drift. Arterial hypertension is the clearest example (*q* = 0.06 linear, *q* < 0.001 spline).

Eleven characteristics reached *q* < 0.05 under all four parametrisations and constitute the most robust findings. Eighteen were significant under none. Both groups are identified in Table 6.

### 3.6. Patterns Among the Characteristics Whose Association Changed

**Figure 4:**
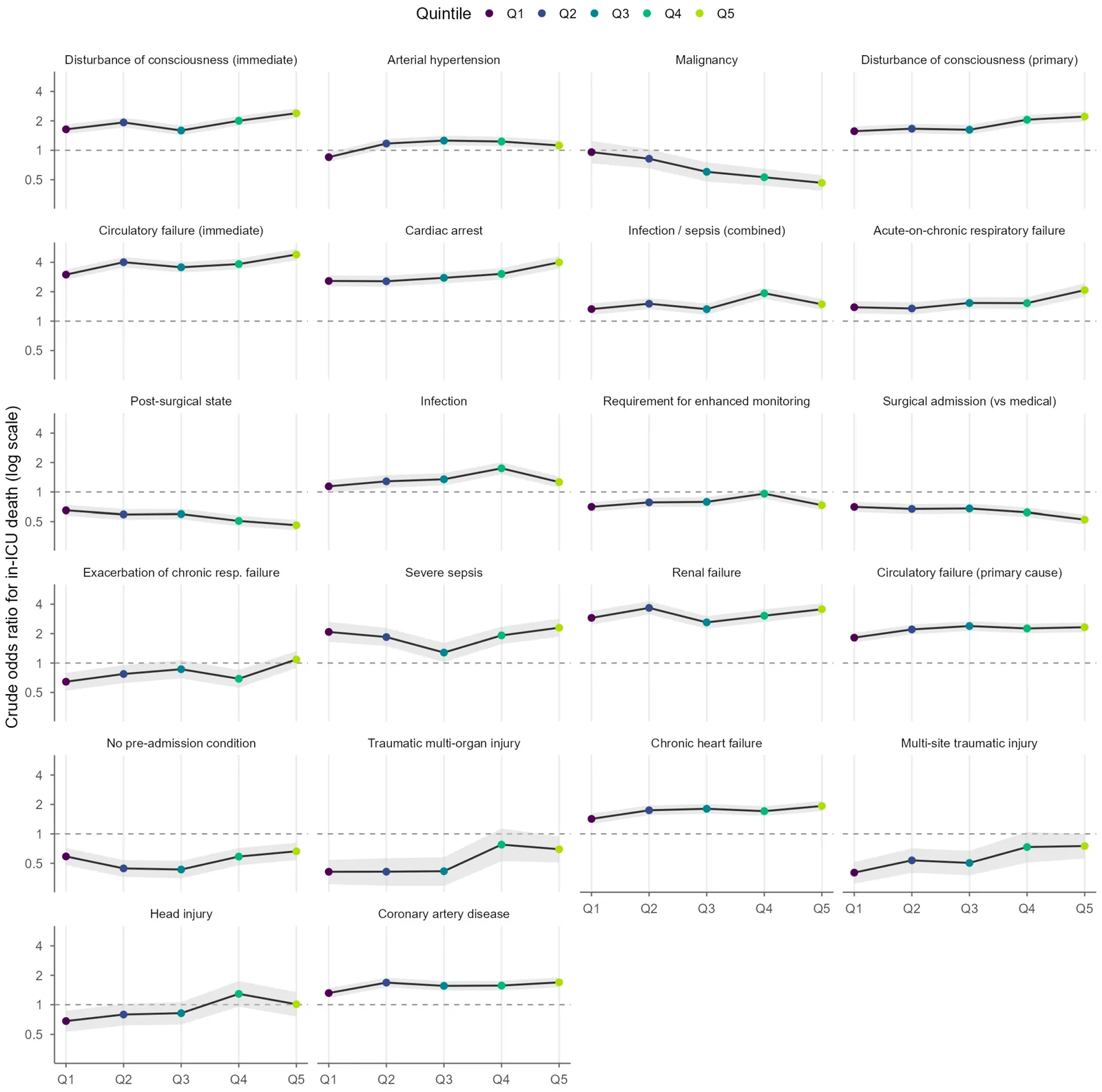
Trajectories of crude odds ratios across the five chronological quintiles for the 22 characteristics with statistically significant temporal heterogeneity (*q* < 0.05, Benjamini–Hochberg). Shaded bands are 95% confidence intervals, the dashed line marks an odds ratio of 1, and the vertical axis is on a common log scale. Characteristics were selected by the formal interaction test, not by inspection. Most trajectories are non-monotonic, which is why a likelihood-ratio test making no monotonicity assumption was preferred to a test for trend.

Among the 22 characteristics with significant temporal heterogeneity, three patterns recur (Figure 4, Table 5). The first is a coherent group of acute cardiovascular, resuscitation-related and neurological states for which the point estimate was higher in Q5 than in Q1, with non-monotonic intermediate values, led by circulatory failure on admission (crude odds ratio 2.99 in Q1 to 4.80 in Q5) and cardiac arrest (2.58 to 4.00). Two chronic cardiovascular comorbidities (chronic heart failure, coronary artery disease) showed the same Q1-to-Q5 direction, as did infection and severe sepsis.

The second pattern is a lower crude odds ratio in Q5 than in Q1 among characteristics that already had a point estimate below one in Q1. Malignancy fell from 0.96 (not significant in Q1) to 0.46, the only monotonic trajectory among the 22. The post-surgical state and surgical admission showed the same Q1-to-Q5 direction.

The third pattern is the loss or reversal of an association with lower odds, most clearly for arterial hypertension, whose point estimate was below 1 in the first quintile (0.85) and above 1 from the second quintile onward, before losing significance again by the last (1.12, 95% CI crossing unity). Exacerbation of chronic respiratory failure (0.64 in Q1 to 1.09 in Q5) and head injury (0.68 to 1.01) crossed unity in the same way. Traumatic multi-organ injury (0.41 to 0.70) and multi-site traumatic injury on admission (0.40 to 0.75) moved in the same direction but remained below 1 in every quintile, an attenuation of the association with lower odds rather than a reversal of it.

### 3.7. Characteristics Without Significant Heterogeneity in the Primary Analysis

Twenty-six of the 48 candidate characteristics did not reach significance in the primary quintile analysis. Failing to reject temporal homogeneity is not the same as demonstrating it, and the following are therefore characteristics for which these data show no heterogeneity rather than characteristics shown to be stable. Several are of interest precisely on that basis. Multi-organ failure—the characteristic with the strongest crude association with death in the earliest quintile—showed no significant heterogeneity in any parametrisation (*q* = 0.27 for quintiles), its crude odds ratio remaining between 2.97 and 4.08 throughout. Age was likewise stable at approximately 1.02 per year in every quintile (*q* = 0.58), as were chronic renal failure, diabetes mellitus, alcoholism, previous CNS stroke, extreme obesity, cachexia and chronic neurological disease. Two characteristics that a descriptive reading of these data would have grouped with the cardiovascular cluster of Section 3.6 require a more careful statement. Neither reaches significance under the primary quintile stratification, but they differ from one another beyond that. Shock on admission (*q* = 0.107 under quintiles) is significant under fixed calendar periods (*q* = 0.022), linear continuous time (*q* = 0.007) and the spline (*q* = 0.037), so it satisfies three of the four parametrisations and is among the better-supported findings under the grading rule of Section 2.7.4, despite failing the primary analysis. Its crude odds ratio rises monotonically from 2.59 to 3.31. Metabolic disturbances on admission (*q* = 0.135 under quintiles) reach significance under fixed calendar periods alone (*q* = 0.038) and are reported as exploratory. Because the primary analysis is the quintile stratification, neither is counted among the 22, and the discrepancy for shock is itself informative about the sensitivity of a stratified test to the choice of strata.

### 3.8. Survival of the Temporal Signal After Adjustment for Case-Mix and for Centre

With centre entered as a fixed effect, 12 of the 48 characteristics retained significant temporal heterogeneity: 11 of the 22 significant in the crude analysis survived, 11 were lost, and one (metabolic disturbances on admission) emerged that the crude analysis had not detected (Table 6). In the balanced panel restricted to the ten centres present in all five quintiles, 10 of 48 reached significance in 62% of the cohort. The eleven characteristics lost after adjustment for centre were coronary artery disease, chronic heart failure, malignancy, the residual “no pre-admission condition” category, circulatory failure as the primary cause of admission, post-surgical state, traumatic multi-organ injury, head injury, surgical admission, renal failure on admission and multi-site traumatic injury on admission. For these, temporal change cannot be separated from which ICUs were contributing at the time.

Six characteristics were significant in the crude analysis and remained significant both after adjustment for case mix (Section 3.9) and after adjustment for centre: disturbance of consciousness on admission, arterial hypertension, circulatory failure on admission, cardiac arrest, infection and severe sepsis. Four of these six were also significant in the balanced panel. For these, the heterogeneity is not explained by the recorded case mix or by centre composition. Whether it reflects a genuine change in prognostic meaning rather than confounding by an unrecorded characteristic cannot be established from these data alone (Section 4.5).

Type of admission, the single characteristic with missing values, required a separate check because its completeness improved over the period. When re-tested with “not recorded” as an explicit third category so that no record was dropped, its temporal heterogeneity remained significant (uncorrected *p* = 0.011, against *p* = 0.003 in the complete-case analysis). Records with an undocumented admission type differed from the remainder (mortality 48.7% versus 43.2%; cardiac arrest 52.2% versus 22.5%), and the changing completeness is stated as a limitation.

### 3.9. Collinearity, Adjusted Model and Temporal Validation

Collinearity among the candidate characteristics was moderate and did not preclude an adjusted model. Of the 1128 pairwise correlations, 15 exceeded 0.4 in absolute value, and the maximum was 0.863. The three highest were composite variables correlated with their own components. The maximum variance inflation factor in an auxiliary model containing all 46 non-composite characteristics was 3.09. None exceeded 5, and there were 239 events per variable (11,009 deaths over 46 characteristics). The rule fixed in advance for fitting an adjusted model was, therefore, met. Within individual quintiles, the maximum variance inflation factor ranged from 2.60 to 5.52, with the only stratum maximum above 5 occurring in the last quintile, where two characteristics exceeded that threshold (Supplementary Table S11).

The adjusted model retained 37 characteristics after removing composites, residual categories and the less informative member of each pair correlated above 0.5 (269 events per variable; that is, 11,009 deaths over 41 model terms). Its discrimination was 0.769 in the whole cohort and improved across quintiles (0.733, 0.767, 0.756, 0.787, 0.799). The quintile terms describe a falling baseline risk of death independent of the recorded characteristics (odds ratio 0.82 for the last quintile relative to the first). Apparent calibration tracked observed mortality closely across the risk distribution, with the largest absolute discrepancy in any decile being 2.9 percentage points. The Hosmer–Lemeshow test was nevertheless significant (*p* < 0.001), as is expected at this sample size, and the full decile table is given in Supplementary Table S12. Re-testing the characteristic-by-quintile interaction inside this model, 15 of 37 characteristics retained significant heterogeneity, and the crude and adjusted analyses agreed on 75.7% of the characteristics tested. Four characteristics significant in the crude analysis lost significance after adjustment, and five gained it—among them multi-organ failure, shock, metabolic disturbances and acute respiratory failure, whose crude stability had concealed adjusted change.

Rolling-origin temporal validation of this model shows where the loss of accuracy over time actually lies. Discrimination did not decay when the model was applied out of period. The areas under the curve on the unseen quintile were 0.759, 0.752, 0.781 and 0.795 (mean 0.772), against an apparent 0.769 on the data used for fitting. The calibration slopes stayed close to unity (0.99 to 1.19), so the fitted weights still applied in a period the model had never seen. What drifted was the level. In the last quintile, the calibration intercept was −0.204, and the model predicted a mortality of 44.4% against an observed 40.5%. The full results are in Supplementary Table S17.

### 3.10. Sensitivity Analyses

Excluding the 921 records with an unconfirmed outcome code (*n* = 24,423) left 24 of 48 characteristics significant and changed the conclusion for 10, the largest of the three movements. This appears largely attributable to compositional change rather than to instability of the method. Those records are concentrated in two centres, and their removal shrinks the last quintile by 15%. Applying the original 180-day length-of-stay rule instead of the 365-day rule (*n* = 25,338) left 22 of 48 significant, with no changed conclusion. Including the pre-2010 historical import excluded from the main cohort (*n* = 25,440) left 23 of 48 significant and changed one conclusion (disseminated atherosclerosis, non-significant in the main cohort at *q* = 0.094, significant at *q* = 0.028 with the import included). The primary result, therefore, does not depend on the choice of date-cleaning rule or on the exclusion of the historical import, but the classification of the unconfirmed-outcome records does affect a minority of the era-specific conclusions.

## 4. Discussion

### 4.1. Principal Findings

Three findings bound what the results in Section 3 can be taken to mean. First, the composition of the cohort changed markedly over the same period, and the number of contributing ICUs fell from 29 to 15 with a corresponding concentration of recruitment. Second, when the same interaction tests are repeated with adjustment for centre, 12 rather than 22 characteristics remain significant, and only six survive adjustment both for centre and for the other recorded characteristics. Third, an adjusted model built on these characteristics does not lose its discrimination when applied to a period it was never fitted on, and its calibration slope stays close to unity. What it loses is the level of predicted risk, over-predicting mortality in the final quintile by about four percentage points. The honest summary is, therefore, narrower than a descriptive reading of these data would suggest. The crude weight of several routinely recorded characteristics does move between eras. A small core of that movement is not explained by the recorded case mix or by centre turnover, and over this horizon the practical consequence for a fitted model is a drift in level rather than a collapse of its internal structure.

### 4.2. What a Moving Crude Odds Ratio Means

Interpreting these movements requires a clear view of what a univariable odds ratio represents. A crude odds ratio is not an intrinsic property of a clinical characteristic but a summary of its association with death within one particular population. It depends jointly on the prevalence of the characteristic and on the composition of the group against which it is contrasted—the remainder of the contemporaneous cohort. A temporal change in a crude odds ratio can, therefore, arise from a genuine change in the lethality of the underlying condition, from a change in the reference population as the case mix and admission practice evolve, or from a change in which patients reach the ICU at all. A purely univariable design cannot distinguish between these on its own. The per-quintile case-mix table makes it possible to examine which is more plausible for a given characteristic [13,19].

Arterial hypertension is the clearest illustration. Its crude association with death reversed direction, from lower odds in the first quintile (0.85) to higher odds thereafter. It is implausible that the pathophysiological consequences of hypertension for a critically ill patient reversed over little more than a decade. Over the same period, the recorded prevalence of hypertension in the cohort rose from 37.5% to 61.1%—the single largest compositional shift in the dataset. As the diagnosis was recorded in a clear majority of admissions rather than a minority, the subgroup it identifies became less selected, and could no longer stand for the comparatively better-preserved, actively managed patients it may have marked in the registry’s early years. The apparent reversal is thus most economically read as confounding by era, and the case-mix table supplies the quantity that makes that reading checkable rather than merely plausible.

Malignancy illustrates the complementary point about centres. Its crude odds ratio moved from 0.96 to 0.46 in a single, monotonic direction across the five quintiles, while its prevalence in the cohort rose from 4.7% to 13.5%. This is consistent with the well-documented improvement in the outcomes of critically ill cancer patients [9]. It is also consistent with something more mundane. The regional oncology ICU increased its contribution over the same period, and when centre is entered as a fixed effect, the temporal heterogeneity of malignancy no longer reaches significance. We cannot separate these two explanations in these data, and we do not claim to. The same applies to the post-surgical state and to surgical admission, which follow the same course and are lost under the same adjustment.

Multi-organ failure makes the opposite point. Its crude odds ratio stayed between 2.97 and 4.08 throughout, and its temporal heterogeneity is not significant under any parametrisation of time. What changed was its rank relative to the acute cardiovascular states, and rank is a relative quantity that moves without any change in the characteristic itself. Ordered lists of risk factors should not be read as fixed hierarchies of lethality, and movement in rank is not, on its own, evidence of a change in risk.

Where the cardiovascular and resuscitation-related associations that were higher in Q5 than in Q1 are concerned, a higher point estimate in the later quintile need not signify a rising absolute risk of death. Over the study period, cardiac arrest as a reason for admission became less frequent (27.1% to 19.9%), while its crude odds ratio was higher in Q5 than in Q1 (2.58 to 4.00), a pattern consistent with the progressive depletion of the more salvageable patients from the ICU-admitted pool: those resuscitated and stabilised without intensive care or admitted under other categories. Circulatory failure on admission, cardiac arrest and disturbance of consciousness on admission are among the six characteristics whose heterogeneity survives adjustment for both case mix and centre, so for these, the compositional explanation is at least incomplete. For the remaining sixteen, it is not possible on these data to say that anything other than the changing population has moved.

### 4.3. Implications for Prognostic Models and for Benchmarking

A model with fixed coefficients derived from a historical development cohort will mis-weight precisely those variables whose association with the outcome has moved. It is useful to separate two phenomena that are often conflated. In level drift, the overall calibration of predicted-to-observed risk shifts while the relative weighting of predictors is preserved. This can be corrected by re-estimating a baseline risk. In structural drift, the coefficients themselves migrate, so that no rescaling of an intercept restores agreement and the model must be re-estimated. It is tempting to assume the second, more demanding form whenever individual associations are shown to drift, but our own temporal validation does not support that reading over this horizon. Out of period, discrimination was preserved and the calibration slopes stayed near unity, while the calibration intercept moved and the model over-predicted death in the final quintile. What these data demonstrate at the level of a fitted model is level drift.

That is a weaker claim than structural drift, but it is not a trivial one. It does not cancel the univariable finding. Twenty-two crude associations, and 15 of 37 adjusted ones, differ significantly between eras. A model is simply more robust to that movement than any single one of its components because the components move partly in compensating directions. Two consequences follow. For prediction, the priority is periodic reassessment of calibration—and re-estimation of baseline risk where it has drifted—rather than wholesale re-derivation, which our data do not show to be necessary on a ten-year horizon in this setting [5,6]. For inference, the consequence is sharper. Any analysis that pools a multi-year registry and reports the association of a single characteristic with death is reporting an average over eras in which that association demonstrably differed. Where the estimand is a single-variable association rather than a prediction, temporal homogeneity should be tested rather than assumed.

This also reframes the long-running debate over the value of mortality prediction in the ICU [4]. The pertinent question is not only whether a score discriminates adequately at a given moment, but whether the population it is applied to is the population on which it was calibrated. The documented divergence between observed mortality in Polish ICUs and that predicted by APACHE II [8] is consistent with exactly this: a local prediction landscape that a static, externally derived model does not fully capture. Benchmarking critical-care outcomes across a decade carries the same risk, since a benchmark silently anchored to an earlier era will mis-rank units for reasons that have nothing to do with their performance.

### 4.4. Comparison with Existing Literature

Our study belongs to a lineage of work built on the Silesian Registry of Anaesthesiology and Intensive Care, whose structure and governance have been described previously [14]. The same database underpins a recent doctoral dissertation that applied predictive scales, machine-learning algorithms and biocybernetic methods to the prognostication of death and complications in intensive care [20]. That work seeks the most accurate predictive model attainable from the registry, whereas the present analysis asks the orthogonal question of whether the univariable associations are themselves temporally stationary.

Several earlier studies from the same setting foreshadow our central finding on a smaller scale. In an analysis of prolonged ventilation after cardiac surgery, prediction models built for two adjacent periods proved to differ from one another, prompting the explicit recommendation that such models be updated whenever the demographics or management of patients change appreciably [21]. At the level of whole scores, mortality in Polish ICUs is substantially lower than the APACHE II system predicts [8], while a local validation of APACHE II, APACHE III and SAPS II in a mixed Polish ICU found acceptable discrimination and calibration at a single point in time [7]—a reminder that cross-sectional validation, however favourable, cannot by construction detect the migration of predictor weights over a decade. The finding that the prognostic importance of individual score components differ between surgical and non-surgical patients and between the sexes [22] reinforces the theme running through our results: the weight a characteristic carries is contingent on the population in which it is measured.

The overall intra-ICU mortality of 43.4% is high by international standards and deserves comment because it bears on the external validity of the absolute figures reported here. Poland has one of the lowest densities of critical-care beds in Europe [23], and Polish ICUs consequently operate under strict admission triage, admitting a strongly selected and more severely ill population than units in health systems with more abundant critical-care capacity. Mortality reported from Polish units is correspondingly higher [8]. Participation in the registry is also voluntary and covers roughly a third of the region’s ICUs, so a further layer of selection cannot be excluded. Absolute mortality in this cohort should, therefore, not be compared directly with that of general critical-care registries elsewhere. The internal, within-registry temporal comparison that is the object of this study is far less sensitive to this because the same selection operates in every stratum, although the accompanying fall in the number of contributing centres means that it is not entirely constant either, which is why the centre-adjusted analysis was performed.

The trends we describe are consistent with well-documented secular changes in the critically ill population reported internationally: mortality attributable to severe sepsis and septic shock declined markedly over comparable years [10], the survival of critically ill patients with cancer improved substantially [9], and the spectrum of diagnoses reaching the ICU has shifted [11], each of which would be expected to alter the association between a baseline characteristic and death. Methodologically, our findings instantiate within a single standardised registry the calibration drift analysed in the prediction-modelling literature [6] and lend weight to dynamic, time-aware modelling approaches [13]. What the present study adds is a formal test applied to every recorded characteristic rather than to a selected few, of whether that drift is visible in the weights of individual, clinically interpretable characteristics, together with an explicit accounting of how much of it survives the two most obvious alternative explanations.

### 4.5. Strengths and Limitations

The principal strength of this study is the resource on which it is based. The Silesian Registry provided a large, multi-centre, regional cohort recorded prospectively over more than nine years under standardised, unchanging variable definitions applied uniformly across participating units. It is precisely this constancy of definitions that makes the central question tractable: the association between each baseline characteristic and death can be compared on a genuine like-for-like basis between chronological periods, which would not be possible in a dataset whose variables were redefined across the study window. Methodologically, the temporal claim rests on a formal interaction test rather than on an inspection of confidence intervals. The test is repeated under four implementations of time. Multiplicity is controlled within each family, and the two leading alternative explanations are tested rather than acknowledged. The entire analysis pipeline asserts its own control numbers at every step and is accompanied by an independent audit of its principal invariants. The code and its execution logs are available from the corresponding author upon reasonable request.

Several limitations temper the interpretation. First, the primary estimand is marginal. The crude odds ratios reported are associations, not adjusted or independent effects, and no causal interpretation is warranted. The adjusted model is supplementary and answers a different question. Second, adjustment for case mix can only use the characteristics the registry records, and it cannot use a physiological severity score. APACHE II scores are absent from the registry, SAPS III scores are available only for a limited subset and SAPS II scores only for a restricted time period. None can therefore serve as a consistent adjustment variable throughout the study period. Residual confounding by unrecorded severity of illness is, therefore, likely, and a characteristic whose heterogeneity survives our adjustment may still be confounded by something we could not measure. Third, centre composition changed substantially, and our two approaches to it fail in opposite directions. Fixed effects over-adjust because centres enter and leave the registry over time, and the fixed effect absorbs part of the genuine temporal signal, while the balanced panel loses power because it discards 38% of the cohort. Their agreement is informative, but neither is definitive, and eleven characteristics fall between them.

Fourth, the quintiles are equal in size rather than in duration, so their calendar spans differ, and statistical power is similar but not identical across strata. This is why fixed calendar periods and continuous time were analysed in parallel, and the three agree on the substance if not on every individual characteristic. Fifth, 921 records (3.6%) carry an outcome code whose exact meaning could not be confirmed with the registry custodian. They are counted as survivors and occur only in the last two quintiles and in two centres; excluding them changes 10 of 48 era-specific conclusions—the largest single source of instability in the analysis. Sixth, type of admission is missing for 5.2% of the records, and its completeness improves over the period. A re-test that retained every record left its temporal heterogeneity significant, but differential completeness remains a source of bias. Seventh, the unit of analysis is the ICU admission, not the patient, because the database carries no reliable identifier across hospitalisations. Re-admissions (5.7% of records) are, therefore, treated as independent. Eighth, registry entries are checked for internal consistency but not routinely audited against the primary medical records, leaving room for misclassification. Ninth, the export supplied to us had been restricted to admissions with a complete clinical record, and we were unable to establish how many incomplete records the registry holds or how they differ, so the selection of the analytical cohort cannot be characterised as fully as reporting standards would require. Finally, the participating units represent about a third of the ICUs in the region, and a small number of records carry an age below 18 years (minimum three years), so the cohort is described as comprising critically ill patients rather than an exclusively adult population. Generalisation beyond the region should be cautious.

### 4.6. Future Directions

Three lines of work follow. The first concetns external validation. The specific characteristics we identify should be re-tested in other long-running ICU registries, since a temporal signal confined to one region and one set of contributing units is of limited value. The second is methodological. Modelling calendar time with time-varying coefficients, rather than testing heterogeneity against a fixed stratification, would characterise the shape and pace of the movement rather than only its existence, and would make better use of the non-monotonic trajectories that dominate our results. The third is operational. The prospective monitoring of individual coefficient weights, alongside the calibration metrics that are already monitored, would allow drift to be detected as it happens rather than reconstructed a decade later.

## 5. Conclusions

In a decade of a large multi-centre ICU registry with unchanging variable definitions, the crude association between a baseline characteristic and intra-ICU death was formally heterogeneous over time for 22 of 48 characteristics, and for 11 under every parametrisation of time examined. Most of these trajectories were not monotonic, so the phenomenon is temporal heterogeneity rather than trend. Much of it tracks a changing population. After adjustment for centre composition, 12 characteristics remain, and 6 survive adjustment for both centre and recorded case mix—circulatory failure, disturbance of consciousness and cardiac arrest among them. For the multivariable model, dataset and time horizon examined in this study, what drifts is the level of predicted risk rather than the relative weighting of the predictors, with discrimination being preserved out of period.

Two practical conclusions follow. Registry-derived risk models and severity scores should undergo periodic reassessment of calibration rather than be treated as permanently valid, and benchmarking of critical-care outcomes across long periods should account for a baseline risk that is itself moving. More importantly for research practice, analyses that pool a multi-year registry to estimate the association of a single characteristic with mortality should test temporal homogeneity rather than assume it because, in these data, that assumption fails for nearly half of the characteristics recorded. The primary estimates reported here are marginal associations from an observational registry and remain hypothesis-generating. The adjusted model of Section 3.9 is a supplementary analysis, and its estimates are conditional. The six characteristics whose heterogeneity survives adjustment for case mix and centre are the appropriate starting point for confirmation elsewhere.

## Figures and Tables

**Table 1:** Baseline characteristics of the analytical cohort, stratified by intra-ICU outcome (*N* = 25,344).

Characteristic	Overall (*N* = 25,344)	Survivors (*n* = 14,335)	Non-Survivors (*n* = 11,009)	*p*
**Demographic and administrative**				
Age, years	66 (56, 76)	64 (53, 74)	69 (59, 78)	<0.001
Female sex	10,595 (41.8)	6005 (41.9)	4590 (41.7)	0.762
Re-hospitalization	1452 (5.7)	915 (6.4)	537 (4.9)	<0.001
Surgical admission (vs medical)	11,374 (47.3)	7134 (52.2)	4240 (40.9)	<0.001
**Pre-admission comorbidities**				
Alcoholism	2283 (9.0)	1193 (8.3)	1090 (9.9)	<0.001
Arterial hypertension	13,191 (52.0)	7305 (51.0)	5886 (53.5)	<0.001
Autoimmune/systemic disease	308 (1.2)	162 (1.1)	146 (1.3)	0.176
Cachexia	901 (3.6)	408 (2.8)	493 (4.5)	<0.001
Chronic heart failure	8804 (34.7)	4216 (29.4)	4588 (41.7)	<0.001
Chronic neurological disease	1952 (7.7)	1163 (8.1)	789 (7.2)	0.005
Chronic renal failure	3718 (14.7)	1634 (11.4)	2084 (18.9)	<0.001
Chronic respiratory failure	3133 (12.4)	1808 (12.6)	1325 (12.0)	0.173
Coronary artery disease	10,439 (41.2)	5238 (36.5)	5201 (47.2)	<0.001
Diabetes mellitus	6361 (25.1)	3346 (23.3)	3015 (27.4)	<0.001
Disseminated atherosclerosis	8505 (33.6)	3893 (27.2)	4612 (41.9)	<0.001
Extreme obesity	1456 (5.7)	810 (5.7)	646 (5.9)	0.478
Malignancy	2122 (8.4)	1434 (10.0)	688 (6.2)	<0.001
No pre-admission condition	2441 (9.6)	1704 (11.9)	737 (6.7)	<0.001
Other pre-admission condition	3781 (14.9)	2156 (15.0)	1625 (14.8)	0.548
Previous CNS stroke	1892 (7.5)	961 (6.7)	931 (8.5)	<0.001
**Primary reason for ICU admission**				
Acute neurological condition	1929 (7.6)	1035 (7.2)	894 (8.1)	0.008
Acute pancreatitis	385 (1.5)	174 (1.2)	211 (1.9)	<0.001
Acute respiratory failure	18,939 (74.7)	10,165 (70.9)	8774 (79.7)	<0.001
Acute or exacerbated chronic respiratory failure (composite)	20,246 (79.9)	11,034 (77.0)	9212 (83.7)	<0.001
Cardiac arrest	6102 (24.1)	2241 (15.6)	3861 (35.1)	<0.001
Circulatory failure (primary cause)	11,865 (46.8)	5531 (38.6)	6334 (57.5)	<0.001
Disturbance of consciousness (primary)	10,019 (39.5)	4791 (33.4)	5228 (47.5)	<0.001
Exacerbation of chronic resp. failure	2026 (8.0)	1247 (8.7)	779 (7.1)	<0.001
Head injury	1191 (4.7)	714 (5.0)	477 (4.3)	0.017
Infection	4818 (19.0)	2449 (17.1)	2369 (21.5)	<0.001
Infection/sepsis (combined)	6117 (24.1)	2994 (20.9)	3123 (28.4)	<0.001
Multi-organ failure	3300 (13.0)	1010 (7.0)	2290 (20.8)	<0.001
Other primary cause	1326 (5.2)	813 (5.7)	513 (4.7)	<0.001
Poisoning	379 (1.5)	287 (2.0)	92 (0.8)	<0.001
Post-surgical state	7774 (30.7)	5165 (36.0)	2609 (23.7)	<0.001
Severe metabolic disturbance	1392 (5.5)	492 (3.4)	900 (8.2)	<0.001
Severe sepsis	1806 (7.1)	764 (5.3)	1042 (9.5)	<0.001
Shock	7613 (30.0)	2924 (20.4)	4689 (42.6)	<0.001
Traumatic multi-organ injury	949 (3.7)	682 (4.8)	267 (2.4)	<0.001
**Clinical status at ICU admission**				
Circulatory failure (immediate)	15,415 (60.8)	6886 (48.0)	8529 (77.5)	<0.001
Disturbance of consciousness (immediate)	13,470 (53.1)	6643 (46.3)	6827 (62.0)	<0.001
Metabolic disturbances	5079 (20.0)	1824 (12.7)	3255 (29.6)	<0.001
Multi-site traumatic injury	1142 (4.5)	800 (5.6)	342 (3.1)	<0.001
Non-invasive ventilation	1191 (4.7)	924 (6.4)	267 (2.4)	<0.001
Other immediate cause	908 (3.6)	555 (3.9)	353 (3.2)	0.005
Renal failure	4666 (18.4)	1593 (11.1)	3073 (27.9)	<0.001
Requirement for enhanced monitoring	13,247 (52.3)	7821 (54.6)	5426 (49.3)	<0.001
Respiratory failure	22,754 (89.8)	12,400 (86.5)	10,354 (94.1)	<0.001

Categorical variables are given as *n* (%) and continuous variables as median (interquartile range). *p* values are from Pearson’s chi-squared test for categorical variables and the Wilcoxon rank-sum test for continuous variables. Type of admission was missing for 1319 records (5.2%). Percentages for that variable are computed on records with a documented value.

**Table 2:** Cohort composition, mortality and centre participation by chronological quintile (*N* = 25,344).

Characteristic	Q1 (*n* = 5068)	Q2 (*n* = 5069)	Q3 (*n* = 5069)	Q4 (*n* = 5069)	Q5 (*n* = 5069)
**Cohort and centres**					
*N*	5068	5069	5069	5069	5069
Deaths	2242	2166	2250	2297	2054
Mortality	44.24%	42.73%	44.39%	45.31%	40.52%
Admission dates	2010-09-03 to 2012-01-13	2012-01-13 to 2013-05-08	2013-05-08 to 2014-12-01	2014-12-01 to 2016-11-16	2016-11-16 to 2020-01-25
Contributing ICUs, *n*	29	25	22	18	15
**Demographic and administrative**					
Age, years	65 [53–75]	66 [55–75]	66 [56–76]	67 [57–76]	67 [57–76]
Female sex	2018 (39.8%)	2155 (42.5%)	2044 (40.3%)	2254 (44.5%)	2124 (41.9%)
Re-hospitalization	312 (6.2%)	308 (6.1%)	304 (6.0%)	265 (5.2%)	263 (5.2%)
Surgical admission (vs medical)	2009 (43.4%)	2234 (46.8%)	2327 (48.2%)	2284 (46.5%)	2520 (51.6%)
**Pre-admission comorbidities**					
Alcoholism	403 (8.0%)	498 (9.8%)	479 (9.4%)	455 (9.0%)	448 (8.8%)
Arterial hypertension	1899 (37.5%)	2609 (51.5%)	2737 (54.0%)	2849 (56.2%)	3097 (61.1%)
Autoimmune/systemic disease	49 (1.0%)	64 (1.3%)	53 (1.0%)	66 (1.3%)	76 (1.5%)
Cachexia	171 (3.4%)	177 (3.5%)	203 (4.0%)	197 (3.9%)	153 (3.0%)
Chronic heart failure	1344 (26.5%)	1957 (38.6%)	1888 (37.2%)	1955 (38.6%)	1660 (32.7%)
Chronic neurological disease	329 (6.5%)	414 (8.2%)	438 (8.6%)	424 (8.4%)	347 (6.8%)
Chronic renal failure	559 (11.0%)	721 (14.2%)	800 (15.8%)	820 (16.2%)	818 (16.1%)
Chronic respiratory failure	505 (10.0%)	689 (13.6%)	624 (12.3%)	648 (12.8%)	667 (13.2%)
Coronary artery disease	1761 (34.7%)	2366 (46.7%)	2406 (47.5%)	2039 (40.2%)	1867 (36.8%)
Diabetes mellitus	944 (18.6%)	1304 (25.7%)	1302 (25.7%)	1384 (27.3%)	1427 (28.2%)
Disseminated atherosclerosis	1322 (26.1%)	1925 (38.0%)	1963 (38.7%)	1763 (34.8%)	1532 (30.2%)
Extreme obesity	214 (4.2%)	328 (6.5%)	258 (5.1%)	296 (5.8%)	360 (7.1%)
Malignancy	236 (4.7%)	345 (6.8%)	367 (7.2%)	488 (9.6%)	686 (13.5%)
No pre-admission condition	472 (9.3%)	553 (10.9%)	498 (9.8%)	451 (8.9%)	467 (9.2%)
Other pre-admission condition	701 (13.8%)	890 (17.6%)	980 (19.3%)	774 (15.3%)	436 (8.6%)
Previous CNS stroke	313 (6.2%)	339 (6.7%)	418 (8.2%)	385 (7.6%)	437 (8.6%)
**Primary reason for ICU admission**					
Acute neurological condition	447 (8.8%)	402 (7.9%)	361 (7.1%)	312 (6.2%)	407 (8.0%)
Acute pancreatitis	98 (1.9%)	76 (1.5%)	63 (1.2%)	72 (1.4%)	76 (1.5%)
Acute respiratory failure	3814 (75.3%)	3771 (74.4%)	3626 (71.5%)	3795 (74.9%)	3933 (77.6%)
Acute or exacerbated chronic respiratory failure (composite)	4113 (81.2%)	4050 (79.9%)	3884 (76.6%)	4047 (79.8%)	4152 (81.9%)
Cardiac arrest	1375 (27.1%)	1283 (25.3%)	1231 (24.3%)	1204 (23.8%)	1009 (19.9%)
Circulatory failure (primary cause)	2267 (44.7%)	2196 (43.3%)	2274 (44.9%)	2368 (46.7%)	2760 (54.4%)
Disturbance of consciousness (primary)	2175 (42.9%)	2110 (41.6%)	1984 (39.1%)	1910 (37.7%)	1840 (36.3%)
Exacerbation of chronic resp. failure	416 (8.2%)	397 (7.8%)	370 (7.3%)	402 (7.9%)	441 (8.7%)
Head injury	301 (5.9%)	267 (5.3%)	244 (4.8%)	173 (3.4%)	206 (4.1%)
Infection	856 (16.9%)	863 (17.0%)	895 (17.7%)	981 (19.4%)	1223 (24.1%)
Infection/sepsis (combined)	1085 (21.4%)	1180 (23.3%)	1157 (22.8%)	1316 (26.0%)	1379 (27.2%)
Multi-organ failure	473 (9.3%)	618 (12.2%)	585 (11.5%)	687 (13.6%)	937 (18.5%)
Other primary cause	434 (8.6%)	278 (5.5%)	263 (5.2%)	192 (3.8%)	159 (3.1%)
Poisoning	98 (1.9%)	83 (1.6%)	72 (1.4%)	77 (1.5%)	49 (1.0%)
Post-surgical state	1289 (25.4%)	1435 (28.3%)	1638 (32.3%)	1520 (30.0%)	1892 (37.3%)
Severe metabolic disturbance	247 (4.9%)	308 (6.1%)	236 (4.7%)	292 (5.8%)	309 (6.1%)
Severe sepsis	307 (6.1%)	375 (7.4%)	323 (6.4%)	416 (8.2%)	385 (7.6%)
Shock	1722 (34.0%)	1452 (28.6%)	1463 (28.9%)	1516 (29.9%)	1460 (28.8%)
Traumatic multi-organ injury	257 (5.1%)	208 (4.1%)	181 (3.6%)	112 (2.2%)	191 (3.8%)
**Clinical status at ICU admission**					
Circulatory failure (immediate)	2915 (57.5%)	2950 (58.2%)	3112 (61.4%)	3180 (62.7%)	3258 (64.3%)
Disturbance of consciousness (immediate)	2870 (56.6%)	2988 (58.9%)	2921 (57.6%)	2636 (52.0%)	2055 (40.5%)
Metabolic disturbances	747 (14.7%)	1118 (22.1%)	1073 (21.2%)	1063 (21.0%)	1078 (21.3%)
Multi-site traumatic injury	330 (6.5%)	237 (4.7%)	232 (4.6%)	129 (2.5%)	214 (4.2%)
Non-invasive ventilation	213 (4.2%)	252 (5.0%)	208 (4.1%)	213 (4.2%)	305 (6.0%)
Other immediate cause	331 (6.5%)	165 (3.3%)	114 (2.2%)	228 (4.5%)	70 (1.4%)
Renal failure	777 (15.3%)	873 (17.2%)	947 (18.7%)	934 (18.4%)	1135 (22.4%)
Requirement for enhanced monitoring	2246 (44.3%)	2853 (56.3%)	3134 (61.8%)	3022 (59.6%)	1992 (39.3%)
Respiratory failure	4550 (89.8%)	4632 (91.4%)	4596 (90.7%)	4583 (90.4%)	4393 (86.7%)

Categorical characteristics are given as *n* (%) within the quintile and continuous characteristics as median [first–third quartile]. Percentages for type of admission are computed on records with a documented value. The full table, including fixed calendar periods, is in Supplementary Tables S1 and S2. APACHE II and SAPS III are not tabulated. The registry stores an unrecorded score as 0 rather than as a missing value, and these scores are absent for 48.8% and 72.1% of the cohort, respectively (Section 4.5), so any summary computed over the stored field mixes recorded scores with absence codes and is not interpretable as a description of severity.

**Table 3:** Participating intensive care units and their admissions by chronological quintile.

Intensive Care Unit	Total	Q1	Q2	Q3	Q4	Q5
Cieszyn	2146	92	515	583	576	380
Katowice (Ochojec)	2143	367	328	362	499	587
Katowice (Ochojec Kardio)	2134	373	376	499	489	397
Bytom (Legionów)	2098	251	372	394	491	590
Tychy	1570	277	218	237	470	368
Katowice (CSK)	1491	294	171	0	261	765
Chorzów (Strzelców Byt)	1466	368	298	315	337	148
Zabrze (SCCS 1)	1400	188	127	180	320	585
Bielsko (Onkologia)	1312	144	175	249	289	455
Rybnik	1071	264	290	315	202	0
Tarnowskie Góry	1046	140	153	257	374	122
Jastrzębie	921	329	330	262	0	0
Bielsko (Wojewódzki)	864	379	366	119	0	0
Jaworzno	831	201	217	235	178	0
Żywiec	831	0	90	209	238	294
Wodzisław	729	170	176	192	191	0
Siemianowice (1 Maja)	471	193	170	108	0	0
Piekary Śl	469	141	190	109	29	0
Rydułtowy	407	81	98	92	119	17
Żory	310	76	108	122	4	0
Oława	301	0	0	0	0	301
Zabrze (SK1)	245	57	141	47	0	0
Bielsko (Wyspiańskiego)	229	137	57	35	0	0
Częstochowa (Mirowska)	186	186	0	0	0	0
Zabrze (SCCS 2)	184	0	34	148	2	0
Racibórz	146	81	65	0	0	0
Pszczyna	127	123	4	0	0	0
Katowice (Rydygiera)	91	91	0	0	0	0
Wrocław Borowska	32	0	0	0	0	32
Wrocław MON	28	0	0	0	0	28
Sosnowiec	23	23	0	0	0	0
Częstochowa (Blachownia)	18	18	0	0	0	0
Zabrze (Biskupice)	17	17	0	0	0	0
Gliwice	7	7	0	0	0	0
**Total admissions**	**25,344**	5068	5069	5069	5069	5069
**Contributing ICUs**	**34**	**29**	**25**	**22**	**18**	**15**

Centres are ordered by total number of admissions contributed to the analytical cohort. A zero indicates that the centre contributed no admissions in that quintile. The corresponding table by fixed calendar period is Supplementary Table S4.

**Table 4:** Crude (univariable) odds ratios for intra-ICU death, whole cohort (*N* = 25,344).

Characteristic	Crude OR	95% CI	*p*
**Demographic and administrative**			
Age (per year)	1.021	1.020–1.023	<0.001
Female sex	0.992	0.943–1.043	0.752
Re-hospitalization	0.752	0.674–0.839	<0.001
Surgical admission (vs medical)	0.633	0.601–0.666	<0.001
**Pre-admission comorbidities**			
Disseminated atherosclerosis	1.934	1.834–2.039	<0.001
Chronic renal failure	1.815	1.692–1.947	<0.001
Chronic heart failure	1.715	1.628–1.807	<0.001
Cachexia	1.600	1.400–1.830	<0.001
Coronary artery disease	1.555	1.479–1.636	<0.001
Previous CNS stroke	1.286	1.171–1.412	<0.001
Diabetes mellitus	1.239	1.170–1.311	<0.001
Alcoholism	1.211	1.111–1.319	<0.001
Autoimmune/systemic disease	1.176	0.938–1.472	0.158
Arterial hypertension	1.106	1.052–1.162	<0.001
Extreme obesity	1.041	0.936–1.158	0.461
Other pre-admission condition	0.978	0.912–1.049	0.536
Chronic respiratory failure	0.948	0.879–1.022	0.167
Chronic neurological disease	0.874	0.796–0.960	0.005
Malignancy	0.600	0.545–0.659	<0.001
No pre-admission condition	0.532	0.486–0.582	<0.001
**Primary reason for ICU admission**			
Multi-organ failure	3.465	3.204–3.750	<0.001
Cardiac arrest	2.915	2.746–3.095	<0.001
Shock	2.895	2.739–3.061	<0.001
Severe metabolic disturbance	2.505	2.239–2.806	<0.001
Circulatory failure (primary cause)	2.157	2.050–2.269	<0.001
Severe sepsis	1.857	1.686–2.046	<0.001
Disturbance of consciousness (primary)	1.802	1.712–1.896	<0.001
Acute respiratory failure	1.610	1.519–1.708	<0.001
Acute pancreatitis	1.590	1.300–1.948	<0.001
Acute or exacerbated chronic respiratory failure (composite)	1.534	1.439–1.635	<0.001
Infection/sepsis (combined)	1.500	1.416–1.589	<0.001
Infection	1.331	1.250–1.417	<0.001
Acute neurological condition	1.136	1.035–1.247	0.007
Head injury	0.864	0.767–0.972	0.016
Other primary cause	0.813	0.725–0.910	<0.001
Exacerbation of chronic resp. failure	0.799	0.728–0.877	<0.001
Post-surgical state	0.551	0.522–0.583	<0.001
Traumatic multi-organ injury	0.498	0.430–0.574	<0.001
Poisoning	0.412	0.324–0.520	<0.001
**Clinical status at ICU admission**			
Circulatory failure (immediate)	3.720	3.520–3.933	<0.001
Renal failure	3.097	2.898–3.311	<0.001
Metabolic disturbances	2.879	2.701–3.070	<0.001
Respiratory failure	2.467	2.250–2.707	<0.001
Disturbance of consciousness (immediate)	1.890	1.797–1.988	<0.001
Other immediate cause	0.823	0.718–0.942	0.005
Requirement for enhanced monitoring	0.809	0.770–0.851	<0.001
Multi-site traumatic injury	0.542	0.476–0.617	<0.001
Non-invasive ventilation	0.361	0.314–0.414	<0.001

Crude odds ratios with 95% confidence intervals from separate univariable binary logistic regression models, one per characteristic. Reference categories are medical admission (for surgical admission) and first ICU admission (for re-hospitalisation). For age, the odds ratio is per additional year. Type of admission was modelled on the 24,025 records with a documented value.

**Table 5:** Crude odds ratios for intra-ICU death within each chronological quintile, with the formal test of temporal heterogeneity.

Characteristic	Q1	Q2	Q3	Q4	Q5	LR χ^2^	q (BH)
**Significant temporal heterogeneity (*q* < 0.05), *n* = 22**							
Disturbance of consciousness (immediate)	1.64	1.93	1.60	2.00	2.39	31.9	<0.001
Malignancy	0.96	0.82	0.60	0.53	0.46	28.4	<0.001
Arterial hypertension	0.85	1.17	1.26	1.23	1.12	29.5	<0.001
Circulatory failure (immediate)	2.99	4.01	3.56	3.83	4.80	28.8	<0.001
Disturbance of consciousness (primary)	1.57	1.66	1.62	2.05	2.21	27.9	<0.001
Cardiac arrest	2.58	2.56	2.77	3.04	4.00	26.6	<0.001
Infection/sepsis (combined)	1.33	1.51	1.33	1.93	1.49	21.7	0.002
Acute or exacerbated chronic respiratory failure (composite)	1.39	1.35	1.53	1.53	2.08	19.6	0.003
Infection	1.14	1.28	1.35	1.75	1.26	19.4	0.003
Post-surgical state	0.65	0.59	0.60	0.51	0.46	19.2	0.003
Requirement for enhanced monitoring	0.70	0.78	0.79	0.96	0.73	17.2	0.008
Surgical admission (vs medical)	0.70	0.67	0.68	0.62	0.52	15.9	0.012
Exacerbation of chronic resp. failure	0.64	0.77	0.86	0.69	1.09	15.8	0.012
Severe sepsis	2.08	1.85	1.28	1.92	2.30	15.3	0.014
Renal failure	2.90	3.67	2.60	3.05	3.55	14.2	0.021
Circulatory failure (primary cause)	1.82	2.21	2.39	2.26	2.33	14.1	0.021
Traumatic multi-organ injury	0.41	0.41	0.41	0.78	0.70	13.7	0.023
No pre-admission condition	0.59	0.44	0.43	0.59	0.66	13.6	0.023
Chronic heart failure	1.42	1.74	1.81	1.71	1.93	13.1	0.026
Multi-site traumatic injury	0.40	0.54	0.50	0.73	0.75	13.2	0.026
Head injury	0.68	0.79	0.82	1.29	1.01	12.3	0.035
Coronary artery disease	1.32	1.68	1.56	1.57	1.69	11.9	0.039
**No significant temporal heterogeneity, *n* = 26**							
Other pre-admission condition	1.05	1.02	0.94	0.79	1.12	10.5	0.068
Disseminated atherosclerosis	1.63	2.09	1.99	1.98	2.01	9.6	0.094
Female sex	1.03	0.94	1.13	0.96	0.90	9.2	0.107
Shock	2.59	2.72	2.91	3.04	3.31	9.1	0.107
Metabolic disturbances	2.38	2.94	2.89	3.07	3.20	8.5	0.135
Other immediate cause	0.92	0.89	0.62	0.63	1.10	8.2	0.143
Non-invasive ventilation	0.31	0.33	0.28	0.46	0.44	8.0	0.154
Other primary cause	0.87	0.87	0.90	0.68	0.55	7.6	0.175
Autoimmune/systemic disease	1.84	1.35	1.21	0.69	1.26	7.3	0.187
Acute neurological condition	1.01	1.00	1.30	1.11	1.34	6.9	0.214
Severe metabolic disturbance	2.21	2.76	2.76	3.02	2.06	6.7	0.219
Multi-organ failure	3.46	3.67	2.97	4.08	3.64	6.1	0.267
Acute respiratory failure	1.55	1.51	1.57	1.71	1.83	5.3	0.351
Acute pancreatitis	1.77	1.42	1.07	2.30	1.56	5.2	0.356
Chronic respiratory failure	0.93	0.98	0.96	0.83	1.07	4.7	0.418
Diabetes mellitus	1.22	1.30	1.37	1.16	1.19	4.5	0.434
Respiratory failure	2.13	2.91	2.42	2.48	2.47	4.0	0.498
Alcoholism	1.17	1.18	1.07	1.36	1.30	3.7	0.530
Poisoning	0.47	0.51	0.33	0.29	0.47	3.6	0.546
Age (per year)	1.02	1.02	1.02	1.02	1.02	3.3	0.576
Re-hospitalization	0.64	0.74	0.84	0.78	0.77	2.7	0.673
Previous CNS stroke	1.27	1.36	1.31	1.13	1.40	2.5	0.696
Extreme obesity	0.97	0.93	1.15	1.12	1.09	2.3	0.728
Cachexia	1.49	1.40	1.71	1.81	1.55	1.9	0.794
Chronic neurological disease	0.87	0.78	0.90	0.88	0.94	1.7	0.802
Chronic renal failure	1.77	1.92	1.76	1.83	1.82	0.7	0.952

Crude odds ratios from univariable logistic models fitted within each quintile (*n* = 5068 to 5069). LR χ^2^ is the likelihood-ratio statistic for the characteristic-by-quintile interaction (4 degrees of freedom), and *q* is the Benjamini–Hochberg-corrected *p* value within this family of 48 tests. Characteristics are ordered by *q*. Corresponding estimates with 95% confidence intervals are in Supplementary Table S18.

**Table 6:** Evidence for temporal heterogeneity under four parametrisations of time and its survival after adjustment for case mix and for centre.

Characteristic	Quintiles	Fixed Periods	Linear Time	Spline Time	*n* of 4	Case-Mix adj.	Centre adj.
Disturbance of consciousness (immediate)	<0.001	<0.001	<0.001	0.002	4	✓	✓
Malignancy	<0.001	<0.001	<0.001	<0.001	4	✓	–
Circulatory failure (immediate)	<0.001	<0.001	<0.001	0.002	4	✓	✓
Disturbance of consciousness (primary)	<0.001	<0.001	<0.001	<0.001	4	n/a	✓
Cardiac arrest	<0.001	<0.001	<0.001	<0.001	4	✓	✓
Acute or exacerbated chronic respiratory failure (composite)	0.003	<0.001	0.001	0.003	4	n/a	✓
Post-surgical state	0.003	0.006	<0.001	0.002	4	✓	–
Surgical admission (vs medical)	0.012	0.002	0.001	0.005	4	n/a	–
Traumatic multi-organ injury	0.023	0.044	0.007	0.033	4	n/a	–
Chronic heart failure	0.026	0.013	0.007	0.016	4	–	–
Multi-site traumatic injury	0.026	0.033	0.007	0.027	4	–	–
Arterial hypertension	<0.001	<0.001	0.060	<0.001	3	✓	✓
Infection	0.003	0.001	0.472	0.001	3	✓	✓
Severe sepsis	0.014	0.015	0.488	0.020	3	✓	✓
Circulatory failure (primary cause)	0.021	0.005	0.084	0.008	3	n/a	–
No pre-admission condition	0.023	0.013	0.098	0.020	3	n/a	–
Coronary artery disease	0.039	0.018	0.116	0.026	3	✓	–
Shock	0.107	0.022	0.007	0.037	3	✓	–
Exacerbation of chronic resp. failure	0.012	0.065	0.017	0.061	2	n/a	✓
Head injury	0.035	0.125	0.017	0.071	2	–	–
Other pre-admission condition	0.068	0.007	0.922	0.028	2	n/a	–
Severe metabolic disturbance	0.219	0.022	0.418	0.019	2	–	–
Infection/sepsis (combined)	0.002	0.164	0.210	0.058	1	n/a	✓
Requirement for enhanced monitoring	0.008	0.164	0.354	0.058	1	–	✓
Renal failure	0.021	0.094	0.526	0.674	1	✓	–
Disseminated atherosclerosis	0.094	0.027	0.187	0.058	1	✓	–
Metabolic disturbances	0.135	0.038	0.072	0.061	1	✓	✓
Autoimmune/systemic disease	0.187	0.066	0.511	0.019	1	–	–
Acute respiratory failure	0.351	<0.001	0.106	0.154	1	✓	–
Respiratory failure	0.498	0.013	0.711	0.295	1	–	–
Female sex	0.107	0.286	0.213	0.299	0	–	–
Other immediate cause	0.143	0.609	0.701	0.201	0	n/a	–
Non-invasive ventilation	0.154	0.525	0.140	0.257	0	–	–
Other primary cause	0.175	0.164	0.075	0.204	0	n/a	–
Acute neurological condition	0.214	0.212	0.168	0.397	0	–	–
Multi-organ failure	0.267	0.967	0.770	0.671	0	✓	–
Acute pancreatitis	0.356	0.241	0.922	0.497	0	–	–
Chronic respiratory failure	0.418	0.820	0.734	0.875	0	–	–
Diabetes mellitus	0.434	0.661	0.374	0.674	0	–	–
Alcoholism	0.530	0.820	0.305	0.674	0	–	–
Poisoning	0.546	0.109	0.511	0.170	0	–	–
Age (per year)	0.576	0.516	0.711	0.674	0	–	–
Re-hospitalization	0.673	0.609	0.511	0.317	0	–	–
Previous CNS stroke	0.696	0.843	0.835	0.551	0	–	–
Extreme obesity	0.728	0.284	0.282	0.263	0	–	–
Cachexia	0.794	0.820	0.778	0.470	0	–	–
Chronic neurological disease	0.802	0.285	0.511	0.726	0	–	–
Chronic renal failure	0.952	0.820	0.835	0.881	0	–	–

Except in the case-mix-adjusted column, *q* values are Benjamini–Hochberg corrected within each family of 48 tests. The four families were corrected independently and never pooled. “*n* of 4” counts the parametrisations in which the characteristic reaches *q* < 0.05. Case-mix adjusted: the characteristic-by-quintile interaction re-tested inside the adjusted model of Section 3.9, which retains 37 characteristics. Those 37 interactions form their own Benjamini–Hochberg family of 37, not 48. “n/a” marks the 11 characteristics excluded from that model: two composite variables, four residual or complement categories, and five removed as the less informative member of a pair correlated above 0.5 (Supplementary Table S12, worksheet selection). Centre adjusted: the same interaction re-tested with centre as a fixed effect. ✓ denotes *q* < 0.05 and – denotes *q* ≥ 0.05. The table lists all 48 characteristics, ordered by the number of parametrisations reaching significance and then by *q*.

## Data Availability

The individual-record data underlying this study are held in the Silesian Registry of Anaesthesiology and Intensive Care and are not publicly available. Although the registry holds no direct patient identifiers, the combination of contributing centre and exact admission and discharge dates is potentially identifying, and the dataset was made available to the investigators for this analysis under terms that do not permit onward distribution. Qualified researchers may request access from the custodian of the registry, the Silesian Branch of the Polish Society of Anaesthesiology and Intensive Therapy, by application to the senior author (Ł.J.K., lkrzych@sum.edu.pl), who will forward the request. Access is subject to the custodian’s approval and to a data-use agreement. Every aggregated output table underlying the numbers reported in this manuscript is provided as Supplementary Material. The analysis code, the locked package environment and the execution logs are available from the corresponding author on reasonable request. One internal file listing the 121 excluded records individually is withheld from publication to preserve the confidentiality described above and is available on request.

## Supplementary Materials

The following supporting information can be downloaded at https://www.mdpi.com/article/10.3390/jcm15187013/s1: Figure S1: crude odds ratios for intra-ICU death within each chronological quintile, all 48 characteristics; Figure S2: movement of rank position across quintiles, presented with an explicit caveat that an ordering of crude odds ratios is not a hierarchy of clinical importance; Table S1: cohort composition by chronological quintile (complete); Table S2: cohort composition by fixed calendar period; Table S3: participating centres by quintile; Table S4: participating centres by fixed calendar period; Table S5: summary by quintile and by period, recruitment concentration and centre turnover; Table S6: interaction tests and crude odds ratios by fixed calendar period; Table S7: agreement between the quintile and fixed-period stratifications; Table S8: interaction with linear continuous time; Table S9: interaction with a natural spline of continuous time; Table S10: correlation matrix of the 48 characteristics; Table S11: variance inflation factors overall and within quintiles; Table S12: adjusted multivariable model with discrimination and calibration; Table S13: crude versus adjusted interaction conclusions; Table S14: sensitivity analyses; Table S15: centre-adjusted and balanced-panel analyses and the characteristics robust to the measured adjustments; Table S16: missingness in type of admission; Table S17: rolling-origin temporal validation; Table S18: crude odds ratios with 95% confidence intervals within each quintile; Table S19: evidence for temporal heterogeneity under the four parametrisations of time for all 48 characteristics.

## Abbreviations

APACHE II	Acute Physiology and Chronic Health Evaluation II
AUROC	Area under the receiver-operating characteristic curve
BH	Benjamini–Hochberg
CI	Confidence interval
FDR	False discovery rate
ICU	Intensive care unit
IQR	Interquartile range
LRT	Likelihood-ratio test
OR	Odds ratio
Q1–Q5	First-to-fifth chronological quintile
SAPS	Simplified Acute Physiology Score
SOFA	Sequential Organ Failure Assessment
STROBE	Strengthening the Reporting of Observational Studies in Epidemiology
VIF	Variance inflation factor
