# Supplementary material for: Temporal Heterogeneity of Factors Associated with Intra-ICU Mortality: A Formal Interaction Analysis of the Multi-Centre Silesian ICU Registry (2010–2020)
Source: J Clin Med. 2026 Sep 10;15(18):7013. doi: 10.3390/jcm15187013 (PMC13607942; doi:10.3390/jcm15187013)
Supplement: Supplementary file 1 [file jcm-15-07013-s001.zip › Supplementary_Figures/Figure_S1_forest_by_quintile.pdf]

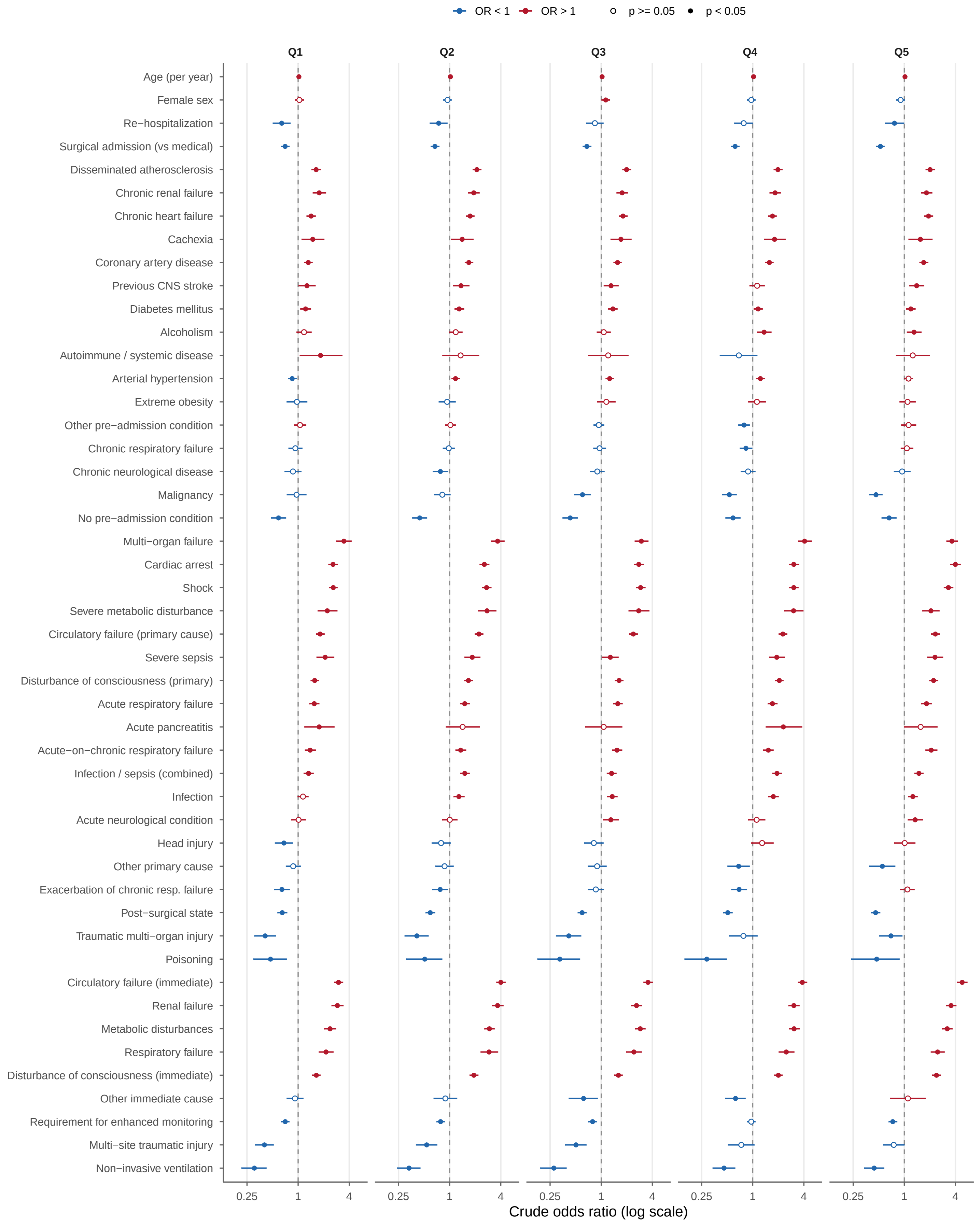

OR < 1
OR > 1
p >= 0.05
p < 0.05
Q1
Q2
Q3
Q4
Q5
Age (per year)
Female sex
Re−hospitalization
Surgical admission (vs medical)
Disseminated atherosclerosis
Chronic renal failure
Chronic heart failure
Cachexia
Coronary artery disease
Previous CNS stroke
Diabetes mellitus
Alcoholism
Autoimmune / systemic disease
Arterial hypertension
Extreme obesity
Other pre−admission condition
Chronic respiratory failure
Chronic neurological disease
Malignancy
No pre−admission condition
Multi−organ failure
Cardiac arrest
Shock
Severe metabolic disturbance
Circulatory failure (primary cause)
Severe sepsis
Disturbance of consciousness (primary)
Acute respiratory failure
Acute pancreatitis
Acute−on−chronic respiratory failure
Infection / sepsis (combined)
Infection
Acute neurological condition
Head injury
Other primary cause
Exacerbation of chronic resp. failure
Post−surgical state
Traumatic multi−organ injury
Poisoning
Circulatory failure (immediate)
Renal failure
Metabolic disturbances
Respiratory failure
Disturbance of consciousness (immediate)
Other immediate cause
Requirement for enhanced monitoring
Multi−site traumatic injury
Non−invasive ventilation
0.25
1
4
Crude odds ratio (log scale)
