# Supplementary material for: Temporal Heterogeneity of Factors Associated with Intra-ICU Mortality: A Formal Interaction Analysis of the Multi-Centre Silesian ICU Registry (2010–2020)
Source: J Clin Med. 2026 Sep 10;15(18):7013. doi: 10.3390/jcm15187013 (PMC13607942; doi:10.3390/jcm15187013)
Supplement: Supplementary file 1 [file jcm-15-07013-s001.zip › Supplementary_Figures/Figure_S2_bump_chart.pdf]

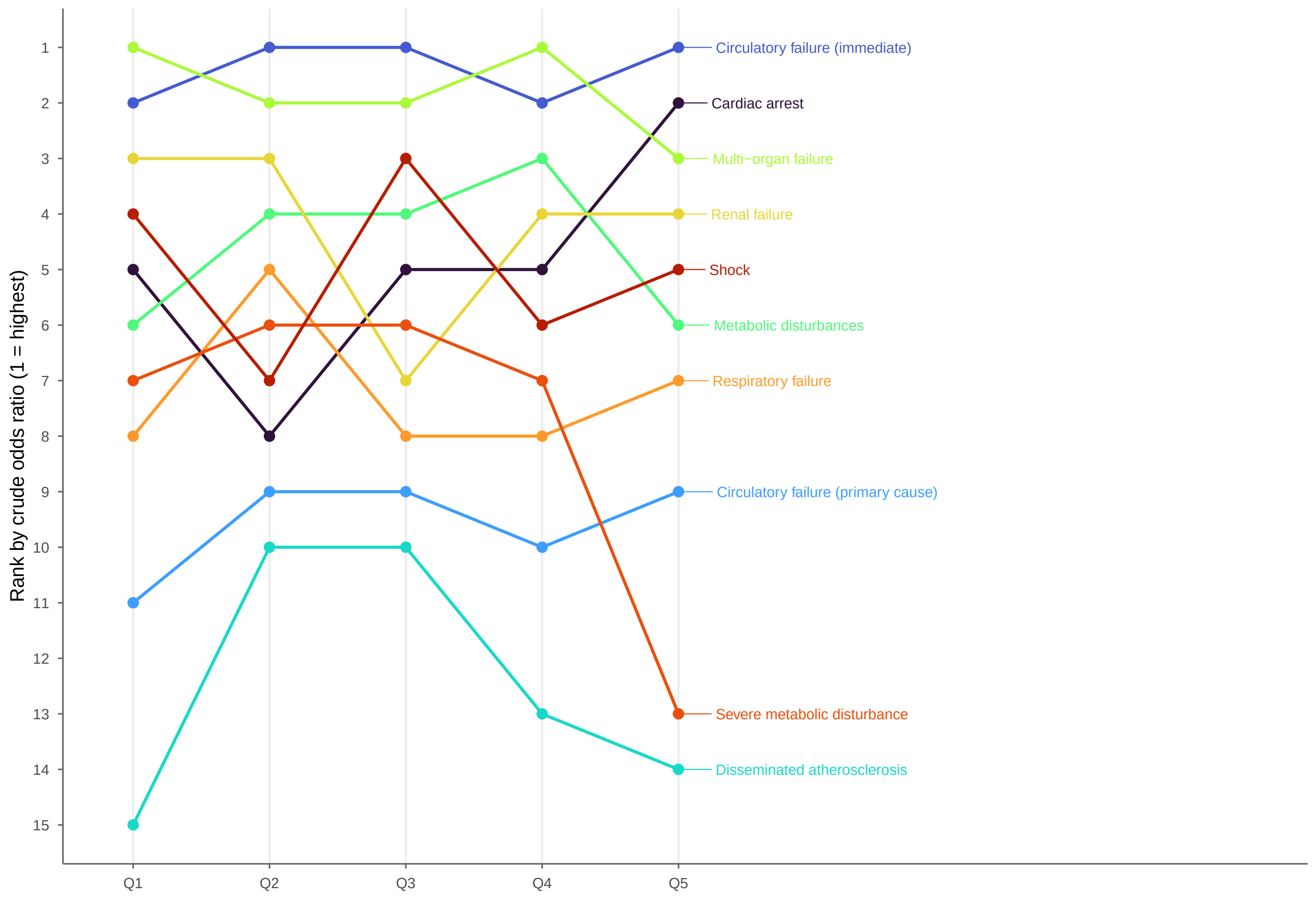

Rank by crude odds ratio (1 = highest)
1
2
3
4
5
6
7
8
9
10
11
12
13
14
15
Q1
Q2
Q3
Q4
Q5
Circulatory failure (immediate)
Cardiac arrest
Multi−organ failure
Renal failure
Shock
Metabolic disturbances
Respiratory failure
Circulatory failure (primary cause)
Severe metabolic disturbance
Disseminated atherosclerosis
